# Mechanochemical Fabrication of Full-Color Luminescent Materials from Aggregation-Induced Emission Prefluorophores for Information Storage and Encryption

**DOI:** 10.1021/jacs.4c02954

**Published:** 2024-06-27

**Authors:** Huilin Xie, Jingchun Wang, Zhenchen Lou, Lianrui Hu, Shinsuke Segawa, Xiaowo Kang, Weijun Wu, Zhi Luo, Ryan T. K. Kwok, Jacky W. Y. Lam, Jianquan Zhang, Ben Zhong Tang

**Affiliations:** † Department of Chemistry, Hong Kong Branch of Chinese National Engineering Research Center for Tissue Restoration and Reconstruction, and Department of Chemical and Biological Engineering, The Hong Kong University of Science and Technology (HKUST), Clear Water Bay, Kowloon, Hong Kong 999077, China; ‡ School of Science and Engineering, Shenzhen Institute of Aggregate Science and Technology, The Chinese University of Hong Kong, Shenzhen (CUHK-Shenzhen), Guangdong 518172, China; § Shanghai Key Laboratory of Green Chemistry and Chemical Processes, Shanghai Frontiers Science Center of Molecule Intelligent Syntheses, School of Chemistry and Molecular Engineering, East China Normal University, 3663 N. Zhongshan Road, Shanghai 200062, China; ∥ Department of Biomedical Engineering, Southern University of Science and Technology (SUSTech), Shenzhen, Guangdong 518055, China

## Abstract

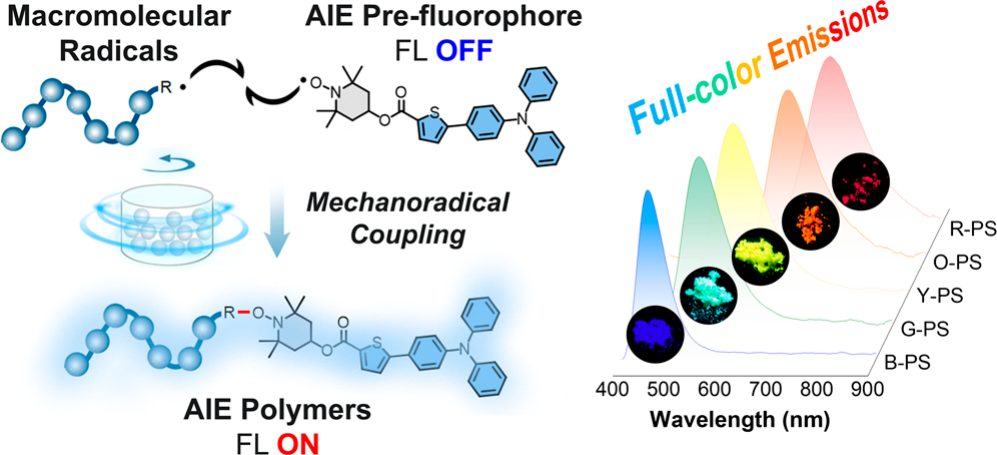

The development of luminescent materials via mechanochemistry embodies a compelling yet intricate frontier within materials science. Herein, we delineate a methodology for the synthesis of brightly luminescent polymers, achieved by the mechanochemical coupling of aggregation-induced emission (AIE) prefluorophores with generic polymers. An array of AIE moieties tethered to the 2,2,6,6-tetramethylpiperidine-1-oxyl (TEMPO) radical are synthesized as prefluorophores, which initially exhibit weak fluorescence due to intramolecular quenching. Remarkably, the mechanical coupling of these prefluorophores with macromolecular radicals, engendered through ball milling of generic polymers, leads to substantial augmentation of fluorescence within the resultant polymers. We meticulously evaluate the tunable emission of the AIE-modified polymers, encompassing an extensive spectrum from the visible to the near-infrared region. This study elucidates the potential of such materials in stimuli-responsive systems with a focus on information storage and encryption displays. By circumventing the complexity inherent to the conventional synthesis of luminescent polymers, this approach contributes a paradigm to the field of AIE-based polymers with implications for advanced technological applications.

## Introduction

Mechanoradicals, engendered by mechanical stimuli, such as grinding, milling, or stretching, offer a distinctive pathway to mechanoradical coupling, facilitating the construction of elaborate molecular architectures.^1−5^ The imposition of mechanical stress upon polymer chains is a well-documented stimulus for the cleavage of covalent bonds, leading to the formation of reactive macromolecular radicals.^4,6−10^ These macromolecular radicals can partake in subsequent reactions with diverse molecules, paving the way for the creation of novel material entities or the initiation of polymerization processes.^11−17^ This burgeoning interest is attributable to the simplicity, cost efficiency, and versatility of the mechanoradical coupling process, coupled with the distinctive properties and wide-ranging applications of the resultant materials.^18−20^ Among these, the synthesis of luminescent polymer materials through mechanoradical coupling represents an especially fascinating domain of exploration.^21−23^ This innovative strategy entails the fusion of nonemissive radical species to forge new compounds that are capable of exhibiting luminescence as a response to mechanical agitation, thus offering a streamlined avenue for the development of functional materials.^22,24,25^

Notwithstanding, the realm of functional luminescent polymer construction through mechanoradical force remains a largely untapped domain.^26^ The primary challenge involves incorporating functional groups into nonspecialized polymers to produce materials with intense emission characteristics, especially in the solid state.^27,28^ Overcoming this obstacle could herald new avenues in the design of advanced polymer materials, bridging the divide between mechanoradical methodologies and functional luminescent applications.^23,25,29,30^ Yet, the process of endowing polymers with fluorescence by utilizing mechanophores is met with several challenges, including complex polymerization demands and the widespread issue of aggregation-caused quenching (ACQ),^31−33^ which markedly dampens emission efficiency when the material is in aggregated or solid states.^34^ The advent of aggregation-induced emission (AIE) luminogens has emerged as a beacon of hope, offering a compelling counter to ACQ ones.^35−38^ AIE luminogens are distinct in that they exhibit intensified emission in the aggregated state, a consequence of restricted intramolecular motions (RIM),^39,40^ which mitigates nonradiative decay pathways.^41,42^ Despite the strides made in embedding AIE luminogens into polymer matrices through blending,^43−46^ copolymerization,^47−49^ or grafting techniques,^50−52^ the exploration of their direct integration via mechanoradical coupling for enhanced luminescence remains in its infancy. This can be attributed to the scarcity of appropriately engineered prefluorophores designed for such processes, as well as the absence of effective and optimized coupling strategies.

In this study, we introduce a material tactic for fabricating luminescent polymers with comprehensive fluorescence by mechanochemically coupling AIE prefluorophores with generic polymers. We synthesized a series of AIE-active moieties linked to the radical compound 2,2,6,6-tetramethylpiperidine-1-oxyl (TEMPO), which initially demonstrated very weak fluorescence due to intramolecular quenching by the existence of radical moieties. However, when embedded in polymer chains through mechanical milling, they are coupled to macromolecular radicals, resulting in significantly enhanced fluorescence. We evaluated the effects of different AIEgens and polymers, finding tunable absorption and emission across the visible-to-near-infrared spectrum. The AIE-modified polymers suggest applications in stimuli-responsive information storage and encryption display. This methodology circumvents complex chemical synthesis, enabling the direct fabrication of highly emissive fluorescent polymers from readily available polymers and AIE prefluorophores. This research furnishes new perspectives on the design of luminescent polymers and applications in information storage, anticounterfeiting, data encryption, and other advanced technologies, heralding substantial implications for the development of polymer material science.

## Results and Discussion

**Figure 1:**
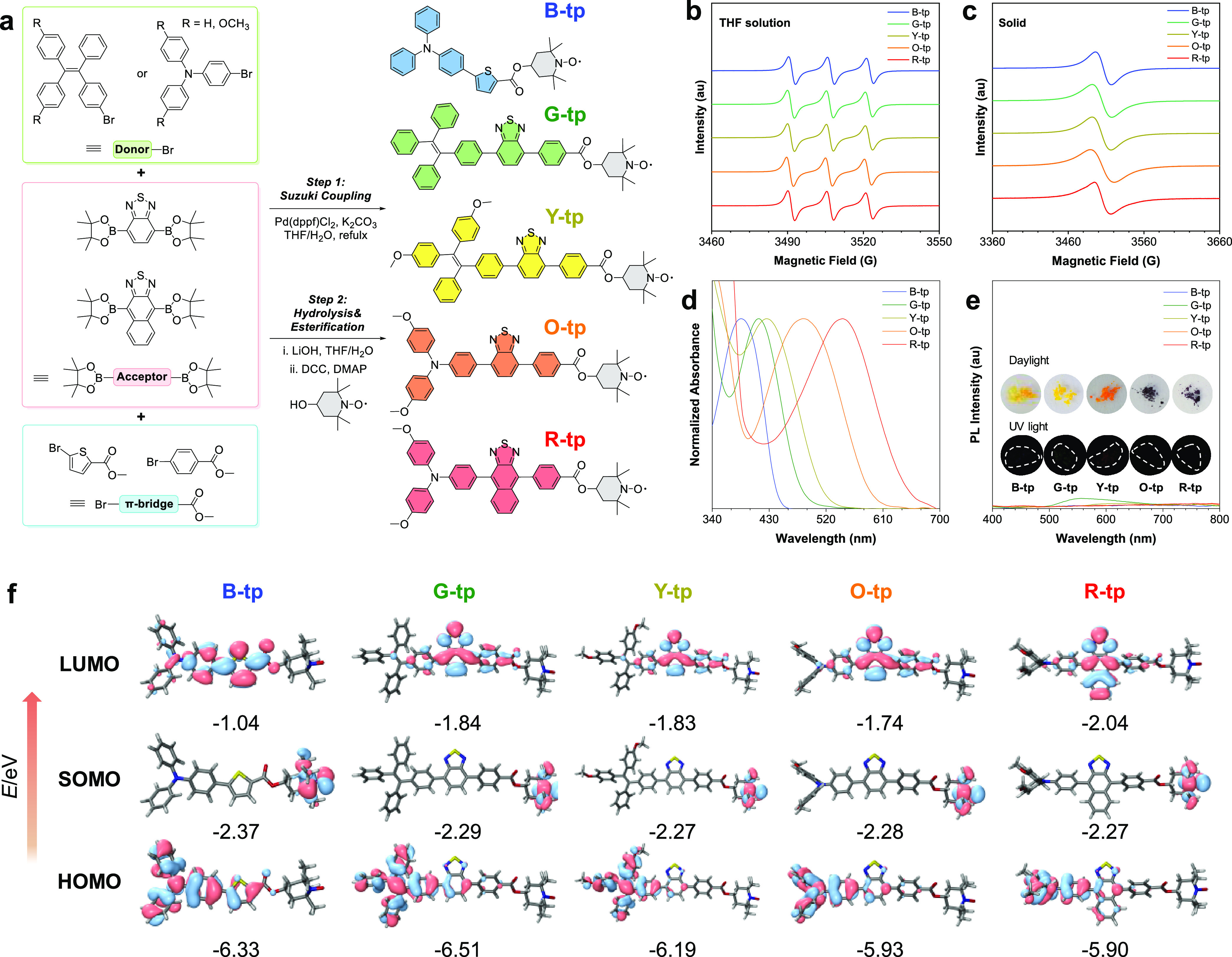
(a) Synthetic route to B-tp, G-tp, Y-tp, O-tp, and R-tp. (b) ESR signal of B-tp, G-tp, Y-tp, O-tp, and R-tp in THF solution, *g* = 1.97. (c) The ESR signal of B-tp, G-tp, Y-tp, O-tp, and R-tp in solid state, *g* = 1.92. (d) Normalized absorbance spectra of B-tp, G-tp, Y-tp, O-tp, and R-tp in the solid state. (e) PL spectra of B-tp, G-tp, Y-tp, O-tp, and R-tp in the solid state. Inset: Corresponding photographs taken under daylight and UV light. (f) LUMOs, SOMOs, and HOMOs of optimized ground-state geometries of B-tp, G-tp, Y-tp, O-tp, and R-tp determined by the M062*X*/6-31G* level of theory.

To achieve materials with emission spanning the entire visible region, a series of AIE prefluorophores with donor–acceptor-π (D-A-π) characteristics were designed (Figure 1a and Scheme S1). The intramolecular charge transfer (ICT) effects of these AIE prefluorophores were carefully modulated by selecting specific donor, acceptor, and π-bridge moieties. The electron-donating groups, such as triphenylamine (TPA) and tetraphenylethylene (TPE) derivatives, were incorporated to confer AIE properties and strong solid-state emission to the molecules. Typical electron-withdrawing building blocks, namely, benzothiadiazole (BT) and naphthothiadiazole (NT), were employed for their established use in organic electronics. The AIE prefluorophores were functionalized with 4-hydroxy-2,2,6,6-tetramethylpiperidine 1-oxyl (TEMPO–OH) through Suzuki coupling, hydrolysis, and Stieglich esterification reactions, aiming to achieve blue (B-tp), green (G-tp), yellow (Y-tp), orange (O-tp), and red (R-tp) emissions. All of the intermediates were confirmed by ^1^H NMR and ^13^C NMR (Figures S1–S10). The final products were determined by matrix-assisted laser desorption ionization-time-of-flight (MALDI-TOF) mass spectrometry, and their purity was examined by high-performance liquid chromatography (HPLC) (Figures S11–S16). The radical signals of the obtained AIE prefluorophores are confirmed by electron spin resonance (ESR) spectroscopy. All of the AIE prefluorophores exhibit strong ESR signals, whether in THF solution (Figure 1b) or in the solid state (Figure 1c), which match well with the signals from TEMPO in the literature.^53,54^ Subsequently, we measured the absorption spectra of the AIE prefluorophores in the solid state. As the ICT increases, the absorption maximum exhibits a gradual red shift, i.e., B-tp, G-tp, Y-tp, O-tp, and R-tp at 386, 413, 427, 484, and 546 nm, respectively (Figure 1d). Interestingly, although all of these AIE prefluorophores have typical AIE moieties, the emissions were very weak, as observed by the naked eye and in the photoluminescence (PL) spectra (Figure 1e).

The molecular configurations and energy levels of the AIE prefluorophores were calculated using density functional theory (DFT) at the M062*X*/6-31G* level (Figure 1f). It is shown that all of the molecules exhibit twisted molecular geometries with large torsion angles among the donor, acceptor, and π-bridges. Additionally, the highest occupied molecular orbitals (HOMOs), the lowest unoccupied molecular orbitals (LUMOs), and the singly occupied molecular orbital (SOMOs) of these molecules are mainly located at the donor, acceptor, and TEMPO parts, respectively. Therefore, the results confirmed the evident HOMO–LUMO separation with gradually decreasing energy gaps, indicating red-shifted absorptions and emissions.

**Figure 2:**
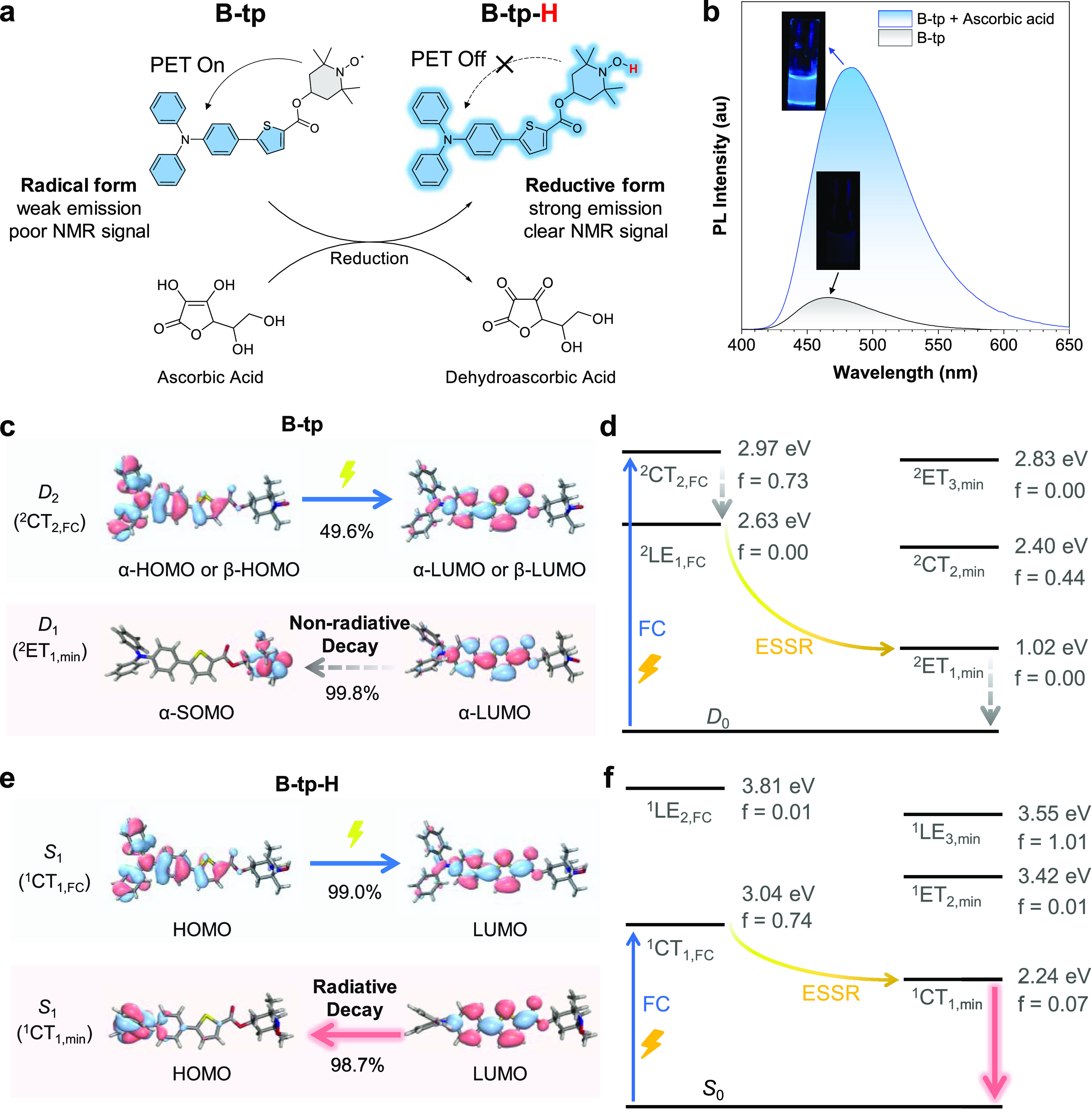
(a) Schematic illustration of the reduction process of B-tp to B-tp-H. (b) PL spectra of B-tp in a THF/water mixture (1:9 vol %) and the turn-on fluorescence after adding ascorbic acid in the same water fraction. (Inset: photos shot before and after adding ascorbic acid to B-tp.) (c) Molecular orbitals for the corresponding electronic transitions at the adsorption *D*_0_ → *D*_2_ (^2^CT_2,FC_) and nonradiative emission *D*_1_ (^2^ET_1,min_) → *D*_0_ processes of B-tp. (d) Calculated energy levels and proposed mechanisms for the luminescent behavior of B-tp. (e) Molecular orbitals for the corresponding electronic transitions at the adsorption *S*_0_ → *S*_1_ (^1^CT_1,FC_) and nonradiative emission *S*_1_ (^1^CT_1,min_) → *S*_0_ processes of B-tp-H. (f) Calculated energy levels and proposed mechanisms for the luminescent behavior of B-tp-H. Blue arrows correspond to absorption, red arrows correspond to emission, and black dash arrows correspond to the nonradiative processes.

To corroborate the molecular architecture and elucidate the luminescent properties of the AIE prefluorophores, we employed ascorbic acid (or vitamin C) as a chemical agent to quench the radical sites. Illustratively, with B-tp serving as a prototype, the nitroxide radical moiety (N–O·) was reduced to a hydroxylamine group (N–OH) in the presence of ascorbic acid, resulting in its reductive form, B-tp-H (Figure 2a). Due to the absence of radicals, B-tp-H demonstrated clear ^1^H NMR signals, with spectral peaks and integrations corroborating the molecular structure of B-tp (Figure S17). Additionally, as depicted in Figure 2b, the B-tp solution, which initially exhibited negligible fluorescence under UV excitation, underwent a dramatic enhancement in luminescence upon treatment with ascorbic acid at room temperature. This manifests a pronounced blue fluorescence and indicates the prompt responsiveness of B-tp to the radical quenching effect of ascorbic acid. Analogously, other members of the AIE prefluorophore series, when treated with ascorbic acid, exhibited significantly enhanced emissions, a testament to the effective quenching of radicals in these systems as well (Figures S18–S21).

To elucidate the underlying causes of the marked differences in fluorescence between B-tp and B-tp-H, we embarked on a computational analysis of their emission mechanisms via time-dependent density functional theory (TD-DFT) at the B3LYP/6-31G* level (Figure 2c). As illustrated in Figure 2d, the predicted optical gap for B-tp is 2.97 eV (*f* = 0.73), which can be ascribed to the *D*_0_–*D*_2_ transition, rather than the *D*_0_–*D*_1_ transition with a negligible oscillator strength (*f* ∼ 0). Moreover, calculations reveal that B-tp readily undergoes a nonradiative transition from *D*_2_ to the *D*_1_ state near the Franck–Condon (FC) conformation right after the absorption process. The transition results in the formation of a *D*_1_ state with local excitation (LE) characteristics, henceforth denoted as ^2^LE_1,FC_, where the superscript indicates the state multiplicity. A subsequent excited state structural relaxation (ESSR) process occurs, leading to the energy minimum conformation of the *D*_1_ state, labeled as ^2^ET_1,min_. This involves an electron transition from the p orbital of the O atom in the TEMPO fragment (p_O_) to the π orbital of the TPA fragment (π_TPA_) (Figures S22–S25), indicative of a photoinduced electron transfer (PET) pathway. The ^2^ET_1,min_ state, generated through ESSR, manifests an oscillator strength approaching 0 to the *D*_0_ state. This transition, from ^2^LE_1,FC_ to ^2^ET_1,min_ via ESSR, effectively quenches radiative decay, thereby accounting for the attenuated fluorescence observed in B-tp. Contrastingly, our computations for B-tp-H reveal a pronounced *S*_0_-*S*_1_ absorption at 3.04 eV (*f* = 0.74, Figure 2e,f). Notably, during ESSR, the electronic state remains invariant, with only geometric alterations occurring, as illustrated in Figures S26 and S27. The *S*_1_-*S*_0_ radiative transition is reasonably strong and fast (2.24 eV, *f* = 0.07), and the intersystem crossing (ISC) quenching pathway from *S*_1_ to *T*_1_ is deemed to be inefficient, given the comparatively insubstantial spin–orbit coupling (SOC) constant of 0.05 cm^–1^. Consequently, B-tp-H molecules are predisposed to fluorescence from the *S*_1_ state. In summary, it is postulated that the diminished fluorescence in B-tp is primarily due to a significant PET effect stemming from the TEMPO radical moiety. Upon annihilation of the radical character via reduction, oxidation, or quenching by another radical, the resultant species should demonstrate considerable “turn-on” fluorescence.

**Figure 3:**
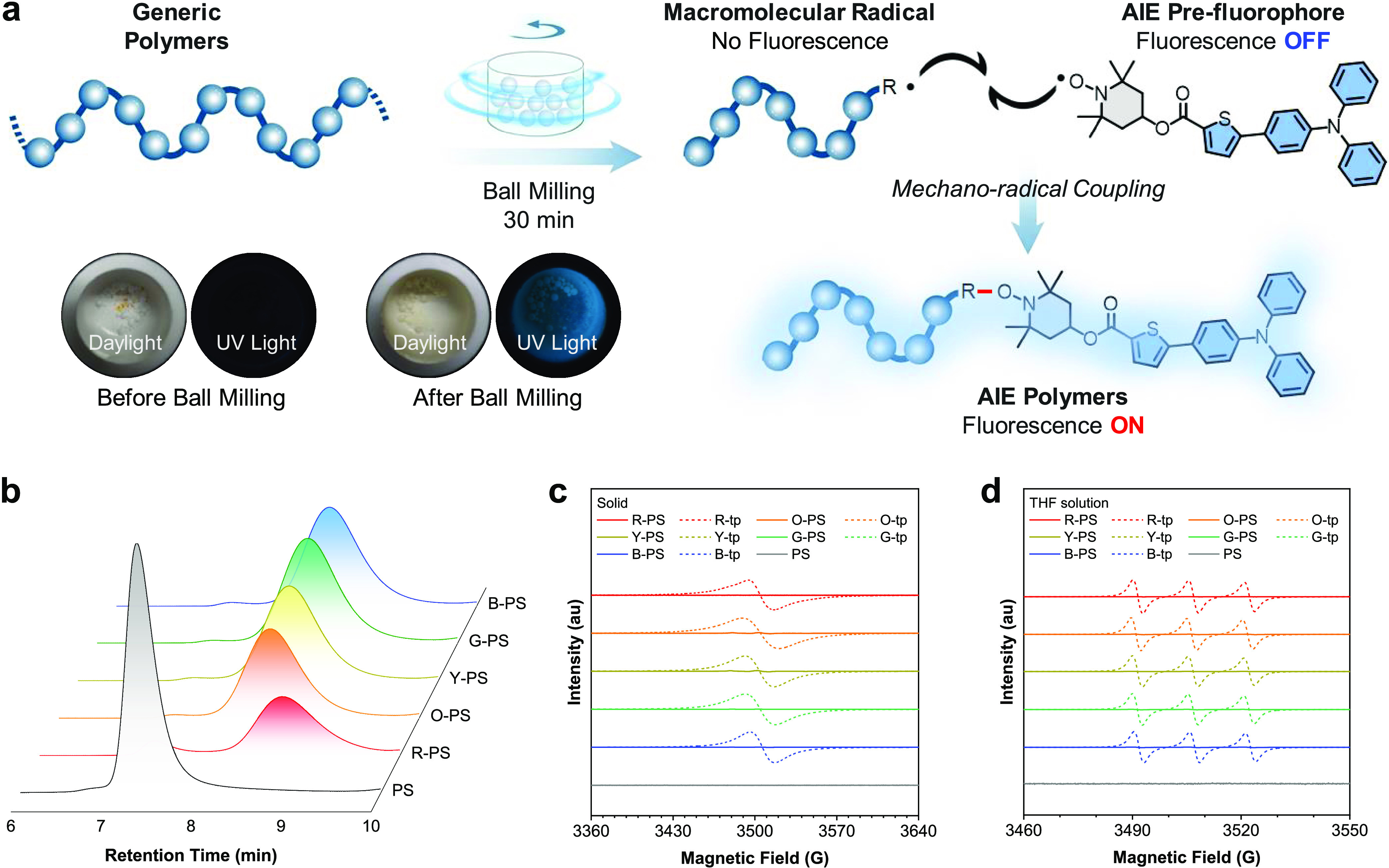
(a) Schematic illustration of the generation of AIE polymers from generic polymers via mechanoradical coupling with AIE prefluorophores, inset: photographs of the mixtures of PS and B-tp before and after ball milling under daylight and UV irradiation. (b) GPC profiles of the original PS and the modified PS (B-PS, G-PS, Y-Ps, O-PS, and R-PS). The ESR signal of B-tp, G-tp, Y-tp, O-tp, R-tp, PS, and the modified PS in (c) solid state (*g* = 1.92) and (d) THF solutions (*g* = 1.97).

Motivated by the viable strategy of mechanochemical coupling, we explored the application of AIE prefluorophores in conjunction with a selection of commercially procured polymers, aiming to validate the practicality of this methodology. Herein, polystyrene (PS), poly(methyl methacrylate) (PMMA), and polyphenylene sulfide (PPS), acquired from Sigma-Aldrich Corp., were utilized as received without any additional purification steps. Upon the exertion of a mechanical stimulus, such as ball milling, scission of the polymer backbones occurs, yielding macromolecular radical intermediates. These emergent radical species from the polymer can engage in covalent interactions with the radical sites of the AIE prefluorophores, leading to the formation of new covalent linkages. This coupling results in the mitigation of the fluorescence quenching effect associated with the free radicals, culminating in the manifestation of intense fluorescence (Figure 3a).

For illustrative purposes, PS with a number-average molecular weight (*M*_n_) of 86.3 kDa and a polydispersity index (PDI) of 1.05 served as a prototype. In the presence of the AIE prefluorophores, B-tp, the PS polymer was subjected to ball milling at a rotational speed of 750 rpm for a duration of 30 min. The resulting mixture, as shown in Figure 3a, displayed a pronounced blue fluorescence upon UV excitation. The reaction products were subsequently subjected to purification procedures involving dialysis and recycling preparative gel permeation chromatography (GPC) to remove any unreacted radicals and other low-molecular-weight residuals, yielding the blue-emissive polymer, denoted B-PS. The synthesis of the additional modified polymers was conducted using analogous protocols. Notably, mechanochemical reactions involving PS derived from various synthetic techniques, such as free-radical polymerization using initiators, have also proven to be successful, highlighting the versatility of this approach (Figure S30). Furthermore, the modified polymers demonstrated excellent photostability and no leakage of the fluorescent dyes, underscoring their robustness for related applications (Figures S31 and S32).

To assess the molecular characteristics of the fluorescently modified PS samples, analytical GPC was performed. As delineated in Figure 3b and detailed in Table S1, a uniform trend was observed across the modified PS samples (B-PS, G-PS, Y-PS, O-PS, and R-PS), where a decrease in the *M*_n_ (10–17 kDa) was accompanied by an increase in the PDI value (1.24–1.25), indicative of the mechanoradical cleavage of homolytic covalent bonds within the PS matrix. The concentration of dyes incorporated into the PS was quantified by constructing calibration curves for the PL intensity of the reduced form of the TEMPO derivatives, plotted against their concentration in dilute solvents (Figure S31).^22^ The results indicated that the dye/PS ratios ranged from 0.020 to 0.032 μmol/mg, corresponding to an incorporation rate of 1.5–1.9% by weight (Table S2). Furthermore, ESR spectroscopy was employed to evaluate the fluorescent polymers. In the solid state (Figure 3c) and in the solution state (Figure 3d), the absence of discernible signals in the ESR spectra signifies the successful neutralization of free radical moieties in the purified fluorescent products after the mechanochemical process. In stark contrast, the original AIE prefluorophores (B-tp, G-tp, Y-tp, O-tp, and R-tp) exhibited distinct ESR signals. Through these observations, we substantiate the transformative mechanochemical coupling as a potent approach for generating luminescent polymers, thereby expanding the potential for innovative applications of AIE materials within the domain of polymer science.

**Figure 4:**
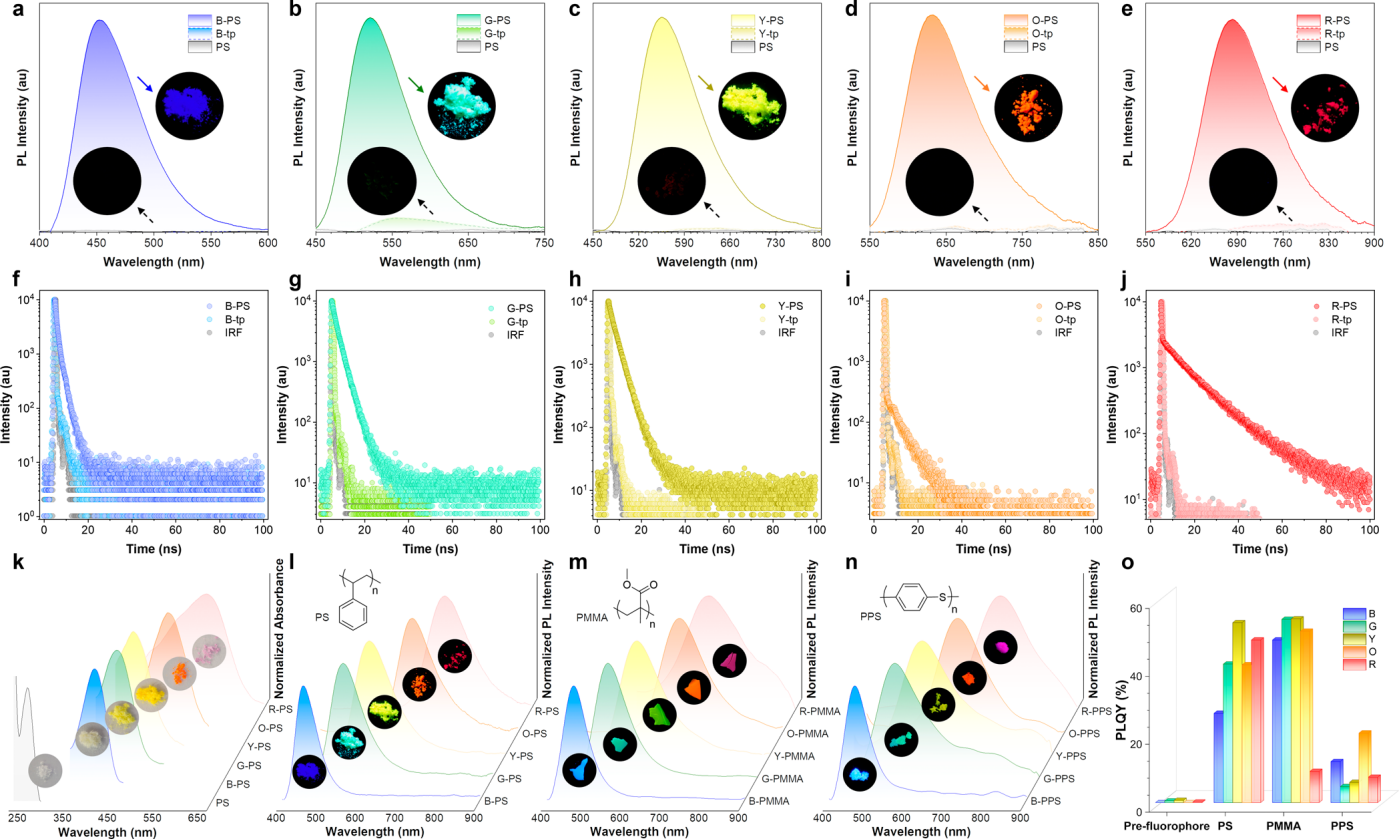
PL spectra of (a) PS, B-tp, and B-PS; (b) PS, G-tp, and G-PS; (c) PS, Y-tp, and Y-PS; (d) PS, O-tp, and O-PS; and (e) PS, R-tp, and R-PS. The PL lifetime profiles of (f) B-tp and B-PS; (g) G-tp and G-PS; (h) Y-tp and Y-PS; (i) O-tp and O-PS; (j) R-tp and R-PS in the solid state. Excitation wavelength: 375 nm. (k) Normalized absorption spectra of PS, B-PS, G-PS, Y-PS, O-PS, and R-PS in the solid state. (l) PL spectra of B-PS, G-PS, Y-PS, O-PS, and R-PS in the solid state. (m) PL spectra of B-PMMA, G-PMMA, Y-PMMA, O-PMMA, and R-PMMA in the solid state. (n) PL spectra of B-PPS, G-PPS, Y-PPS, O-PPS, and R-PPS in the solid state. (o) PLQY of the AIE prefluorophores and the modified polymers.

Subsequent to fabrication of the AIE polymers, an exhaustive assessment of their photophysical properties was performed. First, PL spectra were recorded to elucidate the luminescent properties of the materials (Figure 4a–e). Pure PS was observed to be nonfluorescent, while the AIE prefluorophores alone manifested negligible emissions. However, the modified PS displayed intense fluorescence spanning the entire visible spectrum from blue (G-PS), green (G-PS), yellow (Y-PS), and orange (O-PS) to red (R-PS) emissions. Notably, the emission of the R-PS can extend to the near-infrared (NIR) region. Furthermore, photoluminescence quantum yields (PLQYs) were comprehensively tabulated (Table S3). The data reveal that the AIE prefluorophores exhibited very low PLQYs of <1%. In stark contrast, the modified PS displayed significantly enhanced PLQYs of up to 53.0%, underscoring the efficacious nature of the coupling process in generating highly fluorescent polymers.

The dynamics of the emission for both the AIE prefluorophores and the modified PS were meticulously characterized, with the relevant data depicted in Figure 4f–j. The fluorescence lifetimes (τ_F_) of the AIE prefluorophores were consistently observed to be short, as shown by the comparable signal with the instrument response function (IRF). Significantly, the modified PS exhibited markedly elongated lifetimes in comparison to their AIE prefluorophore counterparts, with recorded lifetimes as follows: τ_F(B-PS)_ = 0.72 ns, τ_F(G-PS)_ = 1.66 ns, τ_F(Y-PS)_ = 2.60 ns, τ_F(O-PS)_ = 6.84 ns, τ_F(R-PS)_ = 12.98 ns, an indication of their intense fluorescence. Moreover, the data reveal a trend, wherein the τ_F_ values of the modified PS were found to extend with the intensification of D–A interactions. This prolongation of τ_F_ is suggestive of more complex excited-state dynamics, possibly involving the transition between the singlet and triplet states. The absorption and emission spectra of the modified PS are summarized in Figure 4k,l, respectively.

To evaluate the adaptability of the synthetic strategy, the AIE prefluorophores were subjected to mechanoradical coupling with PMMA and PPS. The fluorescence profiles of the resultant modified PMMA and PPS were thoroughly investigated, demonstrating desirable absorption and emission characteristics that span the UV to the NIR region, as detailed in Figures 4m,n and S32–S37. Intriguingly, the modified PPS, attributed to their intrinsically richer electronic interactions (i.e., more polar microenvironment) from phenyl rings and sulfur atoms, exhibited emission maxima that were red-shifted relative to their PS and PMMA counterparts when paired with identical AIE prefluorophores. This observation concurs with the anticipated twisted intramolecular charge transfer (TICT) phenomena associated with the AIE chromophores (Figures S38–S42). The PLQYs of these modified PMMA and PPS are also summarized in Figure 4o and Table S2. Similar to the PS cases, the modified PMMA and PPS polymers showcased significantly enhanced PLQYs of up to 54.1% compared with the AIE prefluorophores.

**Figure 5:**
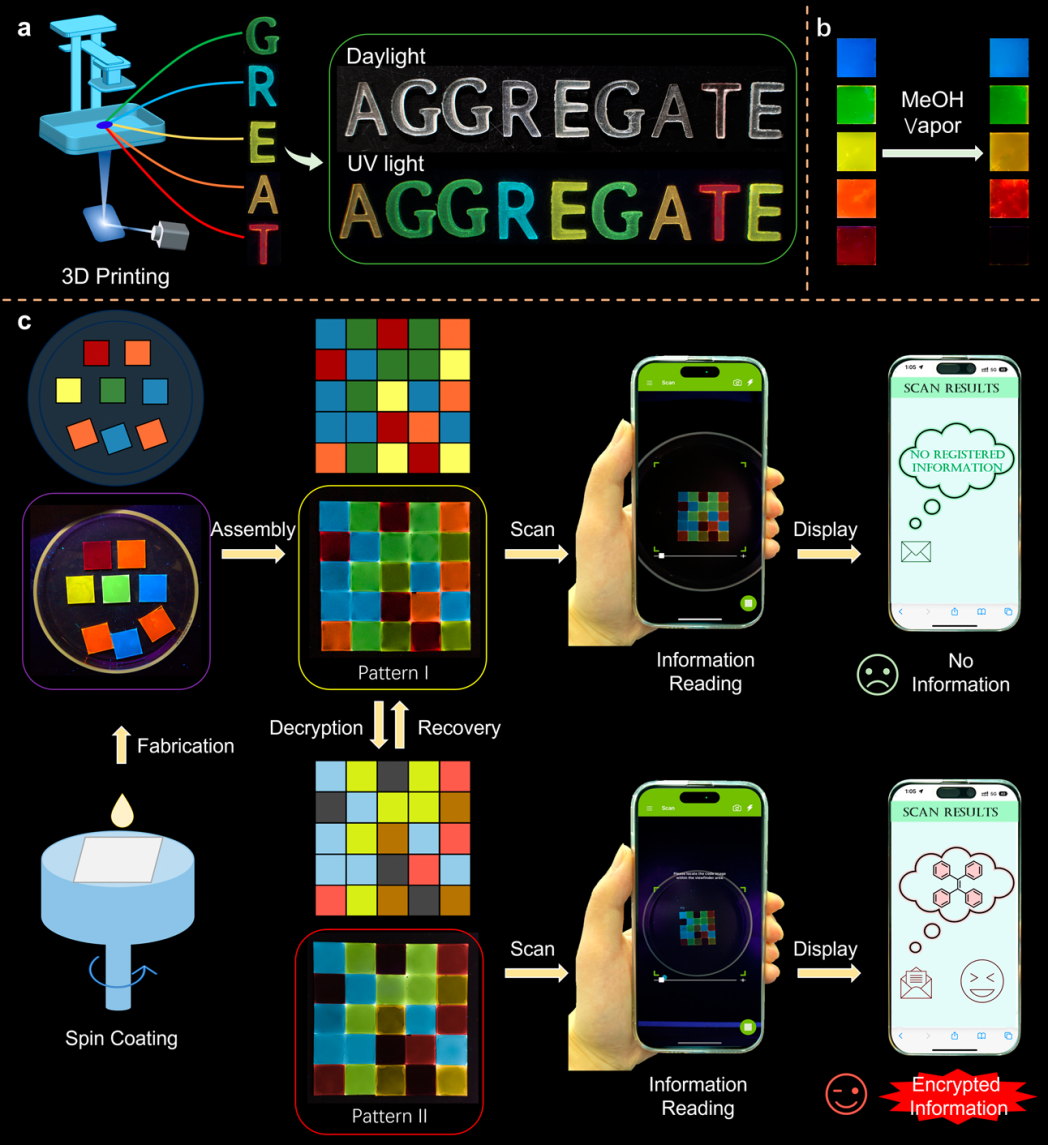
Demonstration of 3D printing and pattern generation for information encryption. (a) Illustration of the 3D printing process and the generation of the fluorescent word “AGGREGATE”. (b) Changes upon methanol-vapor treatment of the fluorescent PS films on silicon wafers. (c) Schematic illustration of fabrication, assembly, writing, and reading of the patterns for dual-mode information encryption.

The fluorescent polymers synthesized herein demonstrate remarkable potential for innovative applications in additive manufacturing, particularly in three-dimensional (3D) printing technologies. To substantiate this assertion, we employed a state-of-the-art digital light processing (DLP) 3D printing apparatus to fabricate a series of individual characters—G, R, E, A, and T, collectively forming the acronym “GREAT.″ As delineated in Figure 5a, each character was engineered to emit a distinct and vibrant color, specifically green, blue, yellow, orange, and red, by using the modified PS materials. The concerted assembly of these characters into the word “AGGREGATE” yielded an intricate display, where the characters appeared homogeneously colored under ambient daylight, yet they revealed their unique fluorescent identities at discrete wavelengths under UV irradiation. This dichotomy of appearances under varying illumination conditions robustly demonstrates the suitability of these fluorescent polymers for 3D printing applications.

Further underscoring the functional versatility of these materials, the polymers exhibited pronounced polarity-responsive fluorescence, a property that can be attributed to the TICT effect inherent to the presenting AIE moieties. As evidenced in Figures S43–S47, this phenomenon manifested as a red shift in emission wavelengths when exposed to polar solvents or vapors. Notably, the magnitude of the observed redshift in emission wavelength was found to be directly correlated with the D–A strength within the AIE moieties. Specifically, it was discerned that an augmentation in the D–A strength elicited a more pronounced red shift in the emission of the polymers. Then, we crafted a series of polymer films on silicon wafers utilizing the spin-coating technique, each designed to display distinct fluorescence. Figure 5b showcases the transformation of these films upon exposure to methanol vapor, the setup of which can be found in Figure S48. The film incorporating Y-PS, initially characterized by bright yellow fluorescence, underwent a shift to orange–yellow fluorescence, while the O-PS film transitioned from orange to red. Most strikingly, the R-PS film, originally emitting in red, experienced a shift extending into the NIR region, thus eluding visual detection. In contrast, the emission of the G-PS film underwent a minor shift from green to yellowish green, whereas the emission of the B-PS film remained unaltered. This compelling demonstration of tunable fluorescence underscores the potential of these AIE polymers to serve as responsive materials for advanced photonic and optoelectronic applications.

Motivated by the dynamic and controllable changes in fluorescence of the modified polymers, we embarked on an endeavor to harness these properties for applications in information encoding and anticounterfeiting measures, as illustrated in Figure 5c. We began by fabricating polymer films and organizing them into a 5 × 5 grid to construct a 3D fluorescent code, designated as pattern I. In its initial state, pattern I did not present any discernible features under ambient daylight and showed only the encoded information when subjected to UV light. The pivotal transformation occurred when pattern I was exposed to methanol vapors. This treatment prompted specific red shifts in the emission wavelength, resulting in the emergence of pattern II. It was at this juncture that the previously concealed data could be decrypted. This hidden information was made legible by scanning the altered pattern with a mobile phone’s camera under UV light exposure. Conversely, any attempts to decipher pattern II using the same scanning process but under natural daylight conditions were unsuccessful, as the embedded information remained obscured. This experimental verification establishes the practicality and effectiveness of a dual-mode fluorescent 3D pattern encrypted through the synergistic application of UV light and solvent vapor interactions. The methodology for crafting such patterns is efficient, drawing on the intrinsic advantages of the modified polymers. These advantages include high PLQYs and outstanding film-forming abilities, which conveniently negate the necessity for external doping agents or the risk of dye leaching. The successful execution underscores the versatility and adaptability of AIE-active materials in security technology, offering a promising avenue for developing sophisticated, customizable encryption systems.

## Conclusions

In this study, we meticulously engineered and synthesized a series of AIE prefluorophores bearing TEMPO radicals as the precursors for mechanoradical coupling. The photophysical properties of these compounds were thoroughly characterized, revealing negligible fluorescence in the solid state. Remarkably, upon radical annihilation, these molecules exhibited a pronounced fluorescence. Experimental and theoretical analyses corroborated the suppression of light emission in the radical state due to the PET effect from the TEMPO moiety. Advancing our methodology, we employed ball milling as a physical strategy for in situ mechanoradical coupling of polymers with AIE prefluorophores, thus covalently embedding AIEgens into the polymer backbone. This approach enabled the facile transformation of nonluminescent polymers, including PS, PMMA, and PPS, into brightly luminescent materials. The resultant modified polymers, benefiting from the inherent AIEgens, demonstrated a long fluorescence lifetime and high PLQYs. Leveraging these advancements, we successfully utilized the resulting polymers in 3D printing applications, achieving high fluorescence intensity with minimal material incorporation. Additionally, the superior film-forming capabilities of the polymers facilitate the creation of films for information storage and encryption. The AIE moieties’ sensitivity to polarity, coupled with UV-exclusive visibility, enabled the formation of dual-mode encrypted 3D fluorescent patterns.

In essence, the intrinsic high PLQYs of AIE materials in the solid state facilitated the efficient “turn-on” fluorescence of the prefluorophores. The mechanoradical coupling technique, requiring no intricate chemical synthesis, demonstrated broad applicability across various prefluorophores and polymers. Consequently, the findings of this research hold substantial promise for the advancement of stimuli-responsive materials and the proliferation of optoelectronic applications. These developments underscore the potential for achieving a comprehensive spectrum of color emissions through strategic structural modification of AIE prefluorophores, with profound implications for both the academic exploration of photophysical mechanisms and the practical development of materials with customized luminescent functionalities.

## Supporting Information Available

The associated content includes synthesis and characterization of the AIE prefluorophores, photophysical characteristics, and theoretical calculations (PDF)
