# Supplementary material for: Mechanochemical Fabrication of Full-Color Luminescent Materials from Aggregation-Induced Emission Prefluorophores for Information Storage and Encryption
Source: J Am Chem Soc. 2024 Jun 27;146(27):18350–9. doi: 10.1021/jacs.4c02954 (PMC11240258; doi:10.1021/jacs.4c02954)
Supplement: Supplementary file 1 — ja4c02954_si_001.pdf [file ja4c02954_si_001.pdf]

Supporting Information

Mechanochemical Fabrication of Full-Color Luminescent Materials from Aggregation-induced Emission Pre-fluorophores for Information Storage and Encryption

Huilin Xie, Jingchun Wang, Zhenchen Lou, Lianrui Hu,* Shinsuke Segawa, Xiaowo Kang, Weijun Wu, Zhi Luo, Ryan T. K. Kwok, Jacky W. Y. Lam, Jianquan Zhang,* and Ben Zhong Tang*

Materials and Methods

Materials

Chemicals were all commercially available without further purification unless noted. All polymers were purchased from Sigma-Aldrich. Solvents for the synthesis were purchased from VWR Chemicals Corp. and used as received.

Characterization

Ball-milling reactions were performed using planetary Ball Mill XQM-04A. UV-vis absorption spectra were recorded on a Varian Cary 50 UV-visible spectrophotometer. Fluorescence spectra were recorded using an Edinburgh FS5 Spectrofluorometer at room temperature. Life-time spectra were recorded using an Edinburgh FLS1000 Spectrofluorometer. The number-average molecular weights (M_n) and molecular weight distributions (M_w/M_ns) were determined by an HLC-8320 GPC. Nuclear magnetic resonance (NMR) spectra were measured on a Bruker AVIII 400 MHz NMR spectrometer. The mass spectrum was obtained on an Xevo G2-XS Top instrument by Water's. The fluorescence quantum yield was obtained on a Hamamatsu UV-NIR absolute PL quantum yield spectrometer C13534. The 3D printing process was proceeded by an Asiga Max™ (X27). For the demonstration of the photo pattern generation, we used a 473 portable UV lamp (excitation wavelength: 365 nm, power: 6 W) from Aiwanke Corp.

Resin preparation and 3D printing

Phenylbis (2,4,6-trimethylbenzoyl)-phosphine oxide (BAPO) weighing 400 mg and equivalent to 0.93 mmol was dissolved in a mixture of 2-hydroxyethyl methacrylate (HEMA) and 2-hydroxyethyl acrylate (HEA) with volumes of 15 mL (0.18 mol) and

19.6 mL (0.18 mol) respectively. Subsequently, 1 mL (3 mmol) of Methacrylic anhydride was added to the resulting solution (referred to as Solution A). Additionally, 10 mg of the fluorescent polymer was dissolved in 1 mL of chlorine dioxide (Solution B). Solution A and Solution B were mixed together and stored in a light-protected environment. A Digital Light Processing (DLP) 3D printing system equipped with a 405-nm LED Wavelength (Asiga Max X27) was used at a temperature of 30 ℃ to fabricate multi-color fluorescent capital characters. The samples were printed with a Z layer thickness of 0.050 mm and an exposure time of 2.7 s, while burn-in layers were exposed for 13 s. To remove any uncured resin, the specimens were immersed in absolute ethyl alcohol and subjected to ultrasound for 10 minutes. Subsequently, they were post-cured at room temperature for 20 minutes using a 405 nm light source.

Mobile Phone Applications. The scan software utilized in this study is COLORCODE® (http://colorzip.com). At the time of publication, this information reading application was accessible for free to conduct code scanning, but the authors are unaware of its availability for any other purpose.

Synthesis

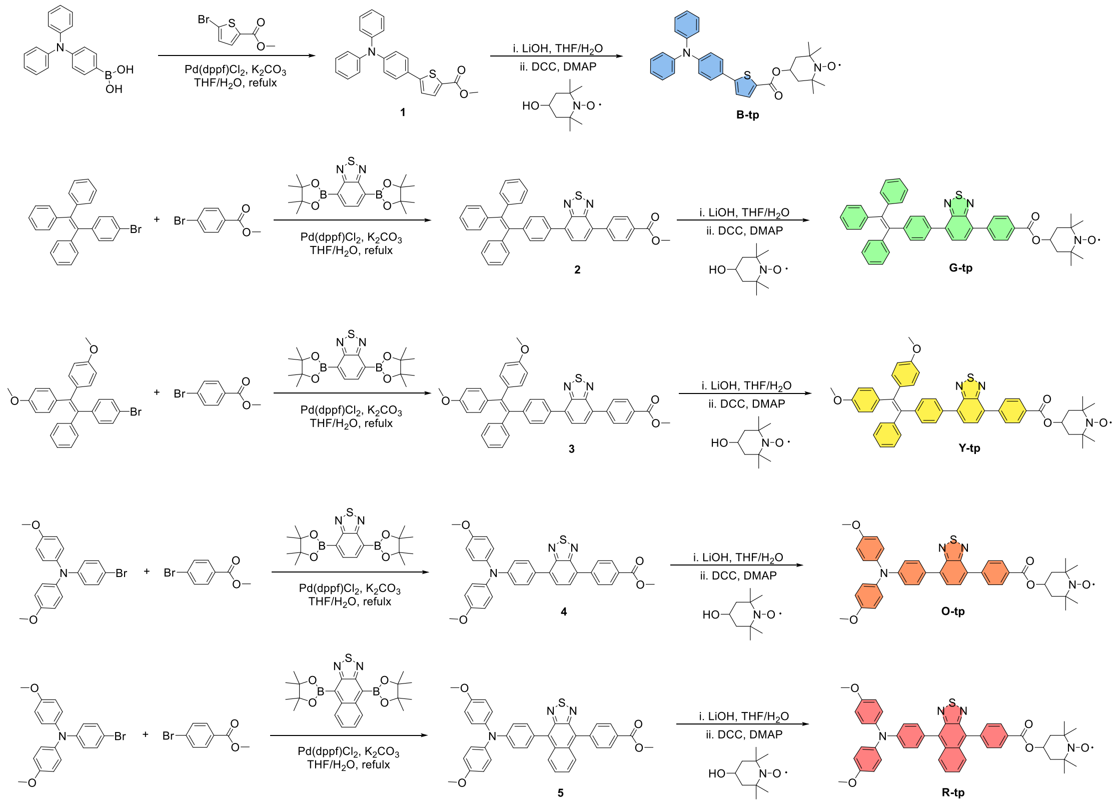

Scheme S1. Synthetic route to B-tp, G-tp, Y-tp, O-tp, and R-tp.

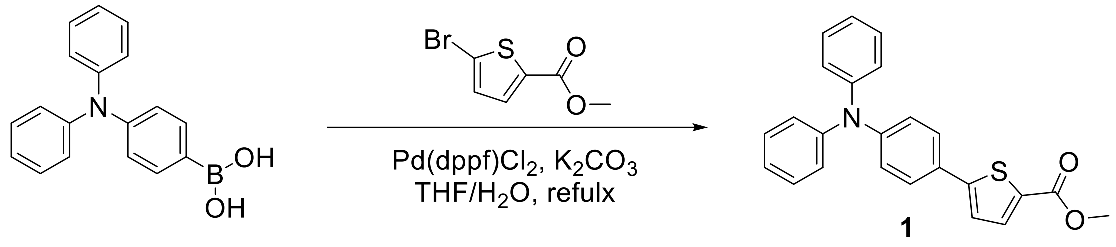

Synthesis of methyl 5-(4-(diphenylamino)phenyl)thiophene-2-carboxylate (1).

A mixture of (4-(diphenylamino)phenyl)boronic acid (1.00 g, 3.46 mmol), methyl 5-bromothiophene-2-carboxylate (913 mg, 4.15 mmol), Pd(dppf)Cl_2 (127 mg, 0.17 mmol) and K_2CO_3 (2.39 g, 17.3 mmol) were dissolved in THF/water (18/9 mL) and stirred at 80 °C overnight under N_2 atmosphere. After being cooled to r.t., the reaction mixture was extracted with chloroform, washed with water and brine. After concentration under reduced pressure, the crude product was purified by column chromatography (stationary phase: silica gel; eluent: DCM) to get the product as light yellow oil (1.16 g, 87%). ^{1}H NMR (400 MHz, $CDCl_3$) δ 7.77 (d, J = 3.9 Hz, 1H), 7.51 (d, J = 8.7 Hz, 2H), 7.31 (t, J = 7.9 Hz, 4H), 7.22 (d, J = 3.9 Hz, 1H), 7.17 (s, 4H), 7.13 – 7.06 (m, 4H), 3.92 (s, 3H). ^{13}C NMR (101 MHz, $CDCl_3$) δ 162.81, 151.41, 148.53, 147.18, 134.59, 130.80, 129.45, 127.00, 126.87, 124.95, 123.61, 122.88, 122.56, 52.14. MS (ESI): calculated for $C_{24}H_{19}NO_2S$: 385.1136, found: 385.1130.

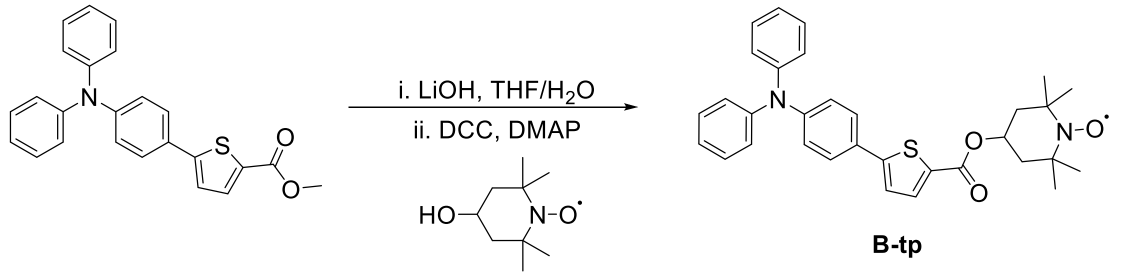

Synthesis of B-tp.

Compound 1 (1.00 g, 2.60 mmol) was dissolved in THF (10 mL), followed by adding aqueous LiOH solution (2M, 20 mL). The mixture was heated at 80 °C overnight, and the reaction was quenched by adding excessive aqueous HCl solution to form

precipitate. The mixture was filtered, and the residue solid was washed with water for three times. The intermediate of carboxylic acid was dried at 60 °C under reduced pressure and used for the next step without further purification. Then, the carboxylic acid, 4-hydroxy-2,2,6,6-tetramethylpiperidin-1-oxyl (TEMPO-OH, 501 mg, 2.91 mmol), N,N′-dicyclohexylcarbodiimide (DCC, 1.00 g, 4.85 mmol), and 4-dimethylaminopyridine (DMAP, 59 mg, 0.49 mmol) were dissolved in anhydrous DCM (20 mL) under N_2 atmosphere. The mixture was stirred at r.t. overnight and purified by column chromatography (stationary phase: silica gel; eluent: DCM to DCM:Et_2O =100:1) to get the product as pale yellow solid (886 mg, 65% over two steps). MS (ESI): calculated for $C_{32}H_{33}N_2O_3S\cdot$: 525.2212, found: 548.2112 (M+Na).

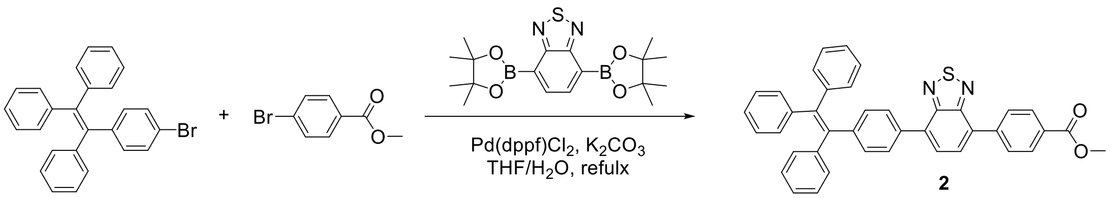

Synthesis of methyl 4-(7-(4-(1,2,2-triphenylvinyl)phenyl)benzo[c][1,2,5]thiadiazol-4-yl)benzoate (2).

A mixture of 4,7-bis(4,4,5,5-tetramethyl-1,3,2-dioxaborolan-2-yl)benzo[c][1,2,5]thiadiazole (1.00 g, 2.58 mmol), methyl 4-bromobenzoate (610 mg, 2.84 mmol), (2-(4-bromophenyl)ethene-1,1,2-triyl)tribenzene (1.17 g, 2.84 mmol), Pd(dppf)Cl_2 (94 mg, 0.13 mmol) and K_2CO_3 (3.56 g, 25.8 mmol) were dissolved in THF/water (26/13 mL) and stirred at 80 °C overnight under N_2 atmosphere. After being cooled to r.t., the reaction mixture was extracted with chloroform, washed with water and brine. After concentration under reduced pressure, the crude product was purified by column chromatography (stationary phase: silica gel; eluent: DCM) to get the product as light yellow solid (650 mg, 42%). ^{1}H NMR (400 MHz, $CDCl_3$) δ 8.23 (d, J = 8.1 Hz, 2H), 8.07 (d, J = 8.1 Hz, 2H), 7.83 – 7.74 (m, 4H), 7.24 (d, J = 8.1 Hz, 2H), 7.20 – 7.11 (m, 13H), 7.08 (dd, J = 7.0, 2.8 Hz, 2H), 3.99 (s, 3H). ^{13}C NMR (101 MHz, $CDCl_3$) δ 166.91, 153.91, 144.10, 143.71, 143.65, 143.61, 141.84, 141.62, 140.45, 135.02, 133.75, 131.83, 131.69, 131.49, 131.40, 131.37, 129.87, 129.71, 129.19,

128.67, 128.46, 127.85, 127.77, 127.67, 126.64, 126.57, 52.26. MS (ESI): calculated for $C_{40}H_{28}N_2O_2S$: 600.1871, found: 600.1864.

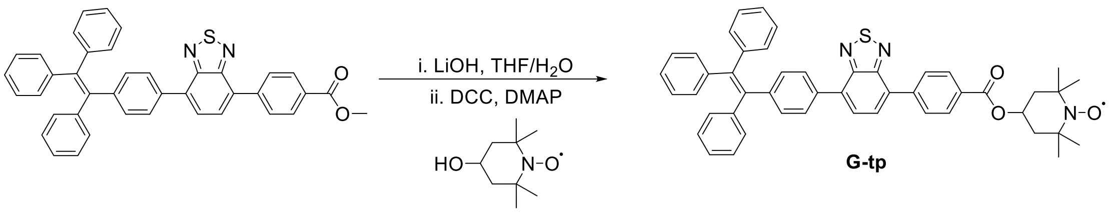

Synthesis of G-tp.

Compound 2 (500 mg, 0.83 mmol) was dissolved in THF (10 mL), followed by adding aqueous LiOH solution (2M, 10 mL). The mixture was heated at 80 °C overnight, and the reaction was quenched by adding excessive aqueous HCl solution to form precipitate. The mixture was filtered, and the residue solid was washed with water for three times. The intermediate of carboxylic acid was dried at 60 °C under reduced pressure and used for the next step without further purification. Then, the carboxylic acid, 4-hydroxy-2,2,6,6-tetramethylpiperidin-1-oxyl (TEMPO-OH, 158 mg, 0.92 mmol), N,N′-dicyclohexylcarbodiimide (DCC, 317 mg, 1.54 mmol), and 4-dimethylaminopyridine (DMAP, 19 mg, 0.15 mmol) were dissolved in anhydrous DCM (10 mL) under N_2 atmosphere. The mixture was stirred at r.t. overnight and purified by column chromatography (stationary phase: silica gel; eluent: DCM to DCM:Et_2O =100:1) to get the product as yellow solid (376 mg, 61% over two steps). MS (ESI): calculated for $C_{48}H_{42}N_3O_3S\cdot$: 740.2947, found: 762.2851 (M+Na).

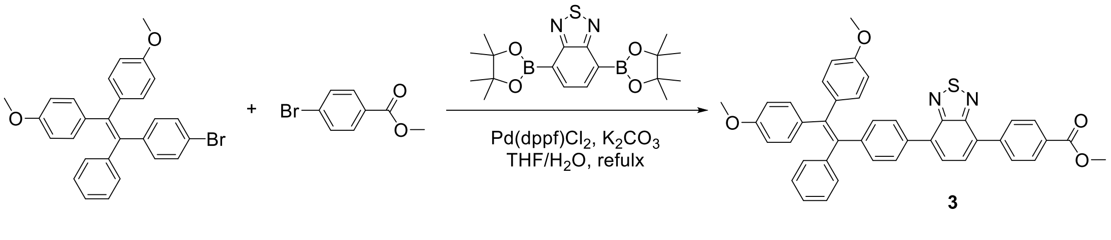

Synthesis of methyl 4-(7-(4-(2,2-bis(4-methoxyphenyl)-1-

phenylvinyl)phenyl)benzo[c][1,2,5]thiadiazol-4-yl)benzoate (3).

A mixture of 4,7-bis(4,4,5,5-tetramethyl-1,3,2-dioxaborolan-2-yl)benzo[c][1,2,5]thiadiazole (1.00 g, 2.58 mmol), methyl 4-bromobenzoate (610 mg, 2.84 mmol), 4,4'-(2-(4-bromophenyl)-2-phenylethene-1,1-diyl)bis(methoxybenzene) (1.33 g, 2.84 mmol), Pd(dppf)Cl_2 (94 mg, 0.13 mmol) and K_2CO_3 (3.56 g, 25.8 mmol) were dissolved in THF/water (26/13 mL) and stirred at 80 °C overnight under N_2 atmosphere. After being cooled to r.t., the reaction mixture was extracted with chloroform, washed with water and brine. After concentration under reduced pressure, the crude product was purified by column chromatography (stationary phase: silica gel; eluent: DCM) to get the product as light orange solid (680 mg, 40%). ^{1}H NMR (400 MHz, $CDCl_3$) δ 8.23 (d, J = 8.4 Hz, 2H), 8.07 (d, J = 8.4 Hz, 2H), 7.85 – 7.75 (m, 4H), 7.22 (d, J = 8.3 Hz, 2H), 7.20 – 7.09 (m, 5H), 7.06 (d, J = 8.7 Hz, 2H), 6.99 (d, J = 8.7 Hz, 2H), 6.69 (dd, J = 12.3, 8.7 Hz, 4H), 3.99 (s, 3H), 3.77 (d, J = 2.3 Hz, 6H). ^{13}C NMR (101 MHz, $CDCl_3$) δ 166.92, 158.26, 158.15, 153.96, 153.93, 144.74, 144.18, 141.87, 140.81, 138.71, 136.33, 136.30, 134.65, 133.84, 132.69, 132.65, 131.72, 131.54, 129.86, 129.69, 129.18, 128.69, 128.48, 127.80, 127.70, 126.23, 113.19, 113.02, 55.13, 55.11, 52.24. MS (ESI): calculated for $C_{42}H_{32}N_2O_4S$: 660.2083, found: 660.2072.

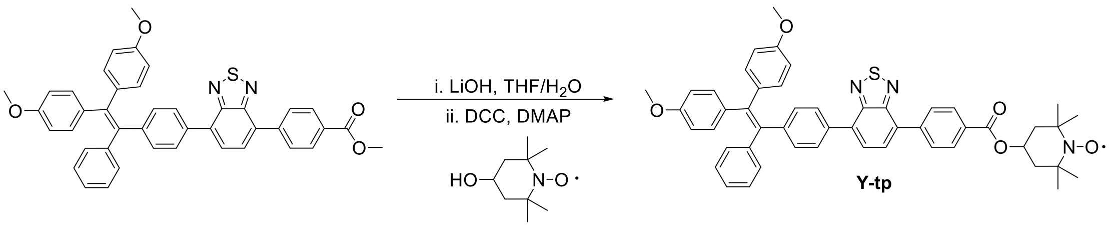

Synthesis of Y-tp.

Compound 3 (500 mg, 0.76 mmol) was dissolved in THF (10 mL), followed by adding aqueous LiOH solution (2M, 10 mL). The mixture was heated at 80 °C overnight, and the reaction was quenched by adding excessive aqueous HCl solution to form precipitate. The mixture was filtered, and the residue solid was washed with water for three times. The intermediate of carboxylic acid was dried at 60 °C under reduced

pressure and used for the next step without further purification. Then, the carboxylic acid, 4-hydroxy-2,2,6,6-tetramethylpiperidin-1-oxyl (TEMPO-OH, 143 mg, 0.84 mmol), N,N′-dicyclohexylcarbodiimide (DCC, 287 mg, 1.39 mmol), and 4-dimethylaminopyridine (DMAP, 17 mg, 0.14 mmol) were dissolved in anhydrous DCM (10 mL) under N_2 atmosphere. The mixture was stirred at r.t. overnight and purified by column chromatography (stationary phase: silica gel; eluent: DCM to DCM:Et_2O =100:1) to get the product as yellow solid (406 mg, 67% over two steps). MS (ESI): calculated for $C_{50}H_{46}N_3O_5S\cdot$: 800.3158, found: 823.3129 (M+Na).

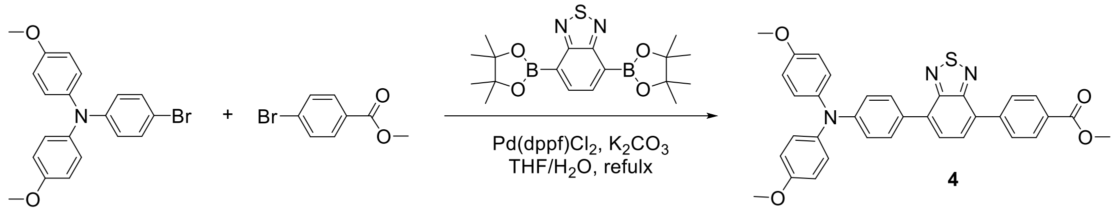

Synthesis of methyl 4-(7-(4-(bis(4-methoxyphenyl)amino)phenyl)benzo[c][1,2,5]thiadiazol-4-yl)benzoate (4).

A mixture of 4,7-bis(4,4,5,5-tetramethyl-1,3,2-dioxaborolan-2-yl)benzo[c][1,2,5]thiadiazole (1.00 g, 2.58 mmol), methyl 4-bromobenzoate (610 mg, 2.84 mmol), 4-bromo-N,N-bis(4-methoxyphenyl)aniline (1.09 g, 2.84 mmol), Pd(dppf)Cl_2 (94 mg, 0.13 mmol) and K_2CO_3 (3.56 g, 25.8 mmol) were dissolved in THF/water (26/13 mL) and stirred at 80 °C overnight under N_2 atmosphere. After being cooled to r.t., the reaction mixture was extracted with chloroform, washed with water and brine. After concentration under reduced pressure, the crude product was purified by column chromatography (stationary phase: silica gel; eluent: DCM) to get the product as light red solid (561 mg, 38%). ^{1}H NMR (400 MHz, $CDCl_3$) δ 8.22 (d, J = 8.0 Hz, 2H), 8.07 (d, J = 8.0 Hz, 2H), 7.85 (d, J = 8.3 Hz, 2H), 7.81 (d, J = 7.5 Hz, 1H), 7.74 (d, J = 7.4 Hz, 1H), 7.17 (d, J = 8.4 Hz, 4H), 7.09 (d, J = 8.3 Hz, 2H), 6.89 (d, J = 8.4 Hz, 4H), 3.99 (s, 3H), 3.84 (s, 6H). ^{13}C NMR (101 MHz, $CDCl_3$) δ 166.93, 156.27, 154.09, 153.96, 149.20, 142.01, 140.43, 133.99, 130.82, 129.89, 129.85, 129.51,

129.10, 128.88, 128.49, 127.15, 126.63, 119.57, 114.82, 55.53, 52.23. MS (ESI): calculated for $C_{34}H_{27}N_3O_4S$: 573.1722, found: 573.1718.

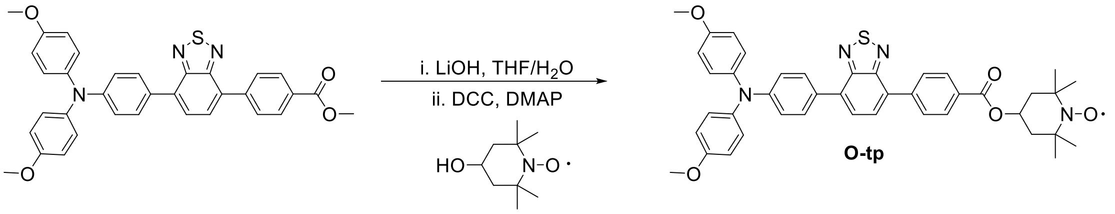

Synthesis of O-tp.

Compound 4 (500 mg, 0.87 mmol) was dissolved in THF (10 mL), followed by adding aqueous LiOH solution (2M, 10 mL). The mixture was heated at 80 °C overnight, and the reaction was quenched by adding excessive aqueous HCl solution to form precipitate. The mixture was filtered, and the residue solid was washed with water for three times. The intermediate of carboxylic acid was dried at 60 °C under reduced pressure and used for the next step without further purification. Then, the carboxylic acid, 4-hydroxy-2,2,6,6-tetramethylpiperidin-1-oxyl (TEMPO-OH, 166 mg, 0.97 mmol), N,N′-dicyclohexylcarbodiimide (DCC, 332 mg, 1.61 mmol), and 4-dimethylaminopyridine (DMAP, 20 mg, 0.16 mmol) were dissolved in anhydrous DCM (10 mL) under N_2 atmosphere. The mixture was stirred at r.t. overnight and purified by column chromatography (stationary phase: silica gel; eluent: DCM to DCM:Et_2O =100:1) to get the product as red solid (386 mg, 62% over two steps). MS (ESI): calculated for $C_{42}H_{41}N_4O_5S\cdot$: 713.2798, found: 736.2698 (M+Na).

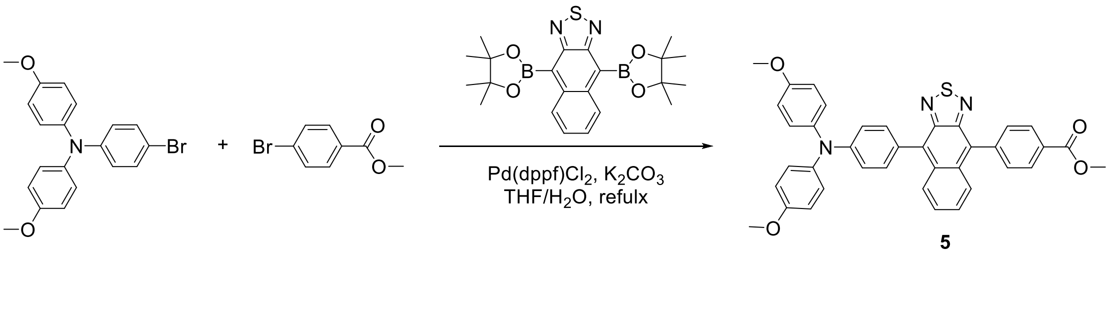

Synthesis of methyl 4-(9-(4-(bis(4-methoxyphenyl)amino)phenyl)naphtho[2,3-c][1,2,5]thiadiazol-4-yl)benzoate (5).

A mixture of 4 4,9-bis(4,4,5,5-tetramethyl-1,3,2-dioxaborolan-2-yl)naphtho[2,3-c][1,2,5]thiadiazole (1.00 g, 2.28 mmol), methyl 4-bromobenzoate (540 mg, 2.51 mmol), 4-bromo-N,N-bis(4-methoxyphenyl)aniline (964 mg, 2.51 mmol), Pd(dppf)Cl_2 (84 mg, 0.11 mmol) and K_2CO_3 (3.15 g, 22.8 mmol) were dissolved in THF/water (22/11 mL) and stirred at 80 °C overnight under N_2 atmosphere. After being cooled to r.t., the reaction mixture was extracted with chloroform, washed with water and brine. After concentration under reduced pressure, the crude product was purified by column chromatography (stationary phase: silica gel; eluent: DCM) to get the product as light purple solid (526 mg, 37%). ^{1}H NMR (400 MHz, $CDCl_3$) δ 8.33 (d, *J* = 8.3 Hz, 2H), 8.25 – 8.18 (m, 1H), 7.94 (ddd, *J* = 7.1, 4.0, 2.2 Hz, 1H), 7.77 (d, *J* = 8.3 Hz, 2H), 7.51 (d, *J* = 8.7 Hz, 2H), 7.43 – 7.34 (m, 2H), 7.26 (d, *J* = 8.9 Hz, 4H), 7.16 (d, *J* = 8.7 Hz, 2H), 6.93 (d, *J* = 9.0 Hz, 4H), 4.03 (s, 3H), 3.85 (s, 6H). ^{13}C NMR (101 MHz, $CDCl_3$) δ 166.96, 156.34, 151.48, 151.22, 148.88, 141.60, 140.46, 132.06, 132.02, 131.78, 131.48, 131.37, 129.79, 129.76, 128.04, 127.64, 127.45, 127.20, 126.85, 126.48, 126.05, 118.78, 114.85, 55.53, 52.31. MS (ESI): calculated for $C_{38}H_{29}N_3O_4S$: 623.1879, found: 623.1872.

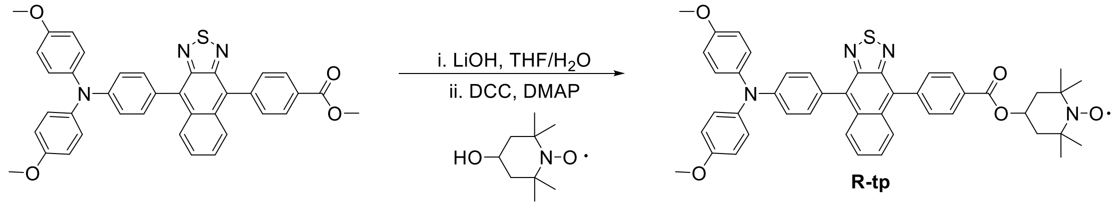

Synthesis of R-tp.

Compound 5 (500 mg, 0.80 mmol) was dissolved in THF (10 mL), followed by adding aqueous LiOH solution (2M, 10 mL). The mixture was heated at 80 °C overnight, and the reaction was quenched by adding excessive aqueous HCl solution to form precipitate. The mixture was filtered, and the residue solid was washed with water for

three times. The intermediate of carboxylic acid was dried at 60 °C under reduced pressure and used for the next step without further purification. Then, the carboxylic acid, 4-hydroxy-2,2,6,6-tetramethylpiperidin-1-oxyl (TEMPO-OH, 152 mg, 0.89 mmol), N,N′-dicyclohexylcarbodiimide (DCC, 304 mg, 1.48 mmol), and 4-dimethylaminopyridine (DMAP, 18 mg, 0.15 mmol) were dissolved in anhydrous DCM (10 mL) under N_2 atmosphere. The mixture was stirred at r.t. overnight and purified by column chromatography (stationary phase: silica gel; eluent: DCM to DCM:Et_2O =100:1) to get the product as purple solid (336 mg, 55% over two steps). MS (ESI): calculated for $C_{46}H_{43}N_4O_5S\cdot$: 763.2954, found: 763.2960.

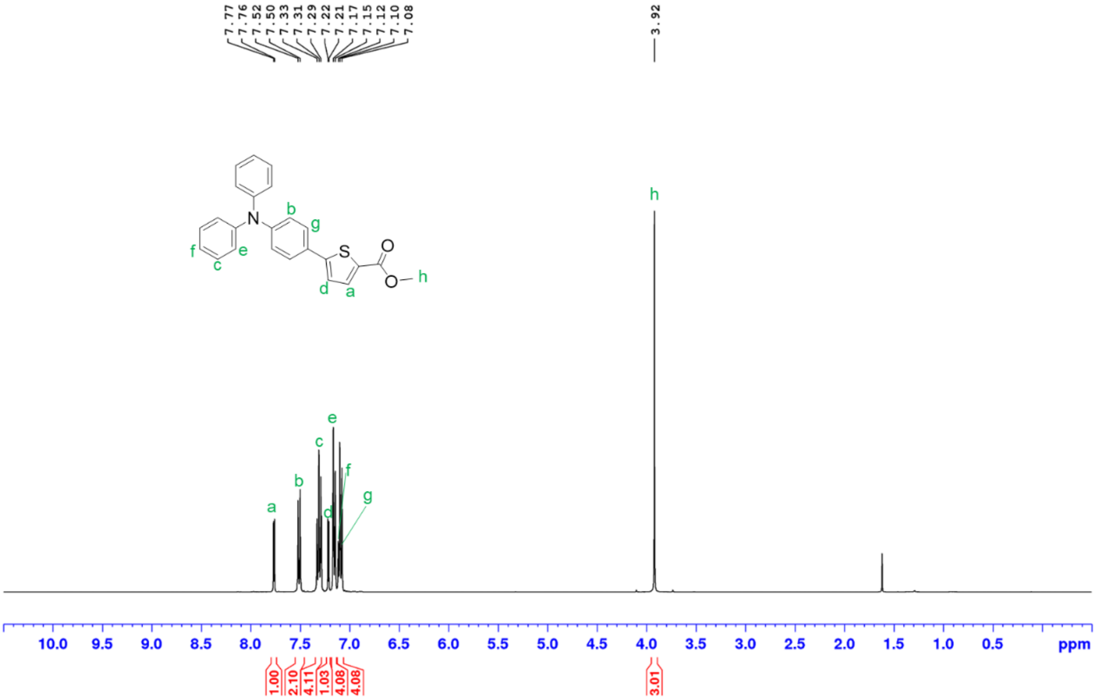

Figure S1. ^{1}H NMR spectrum ($CDCl_3$, 400 MHz, 298 K) of compound **1**.

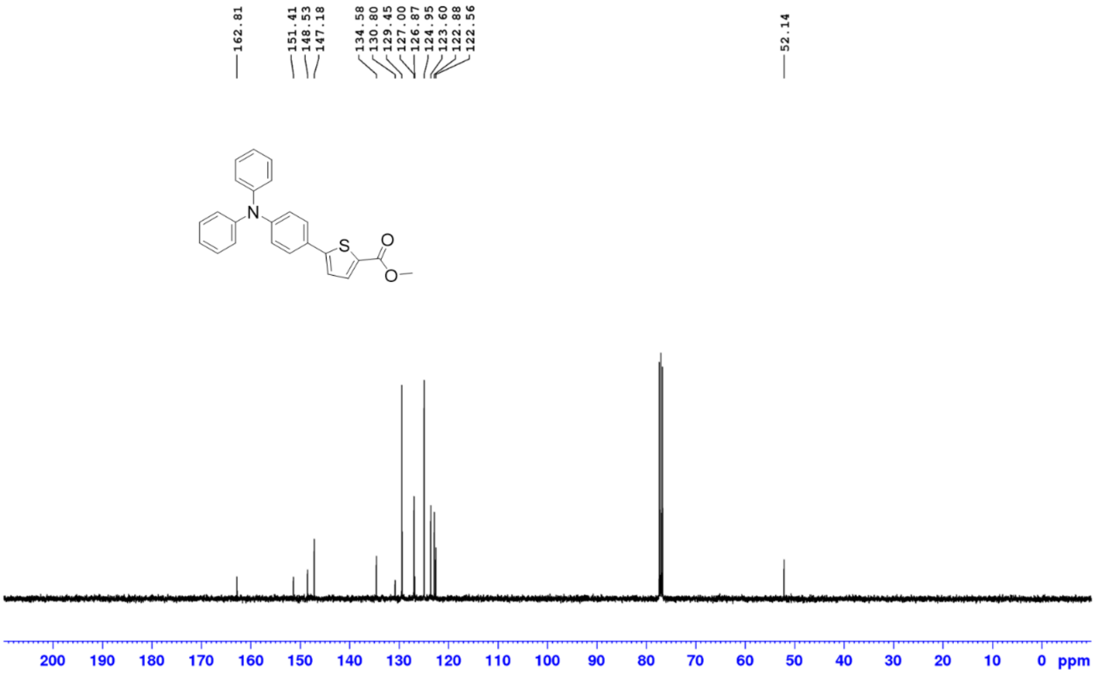

Figure S2. ^{13}C NMR spectrum ($CDCl_3$, 100 MHz, 298 K) of compound **1**.

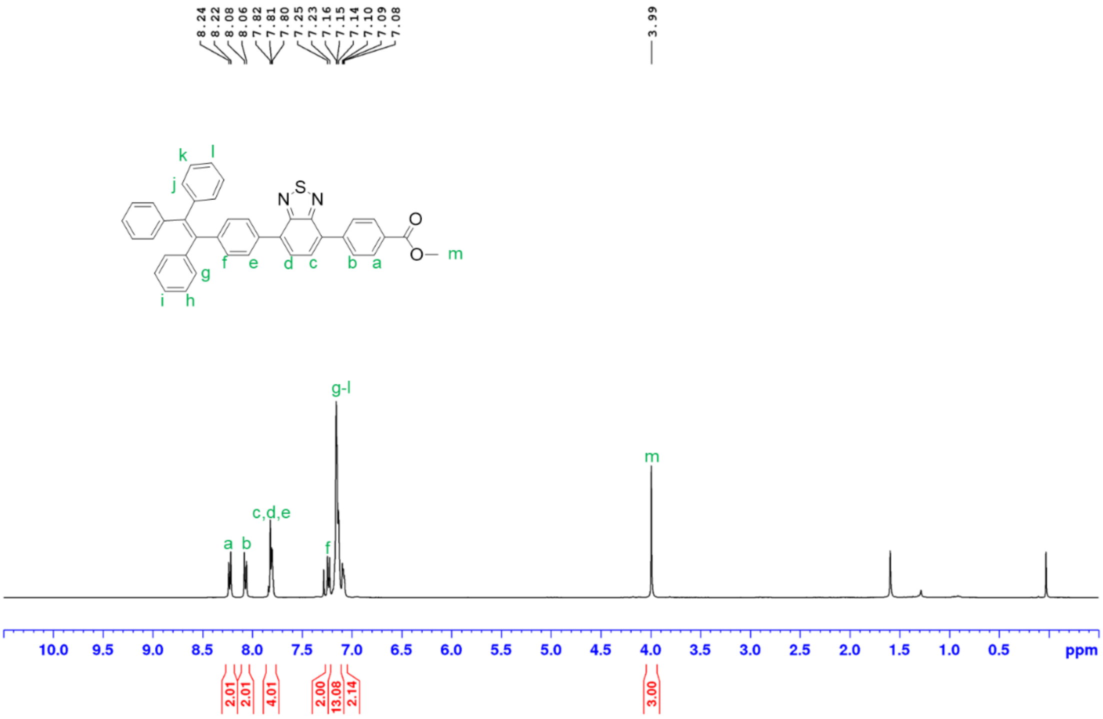

Figure S3. ^{1}H NMR spectrum ($CDCl_3$, 400 MHz, 298 K) of compound **2**.

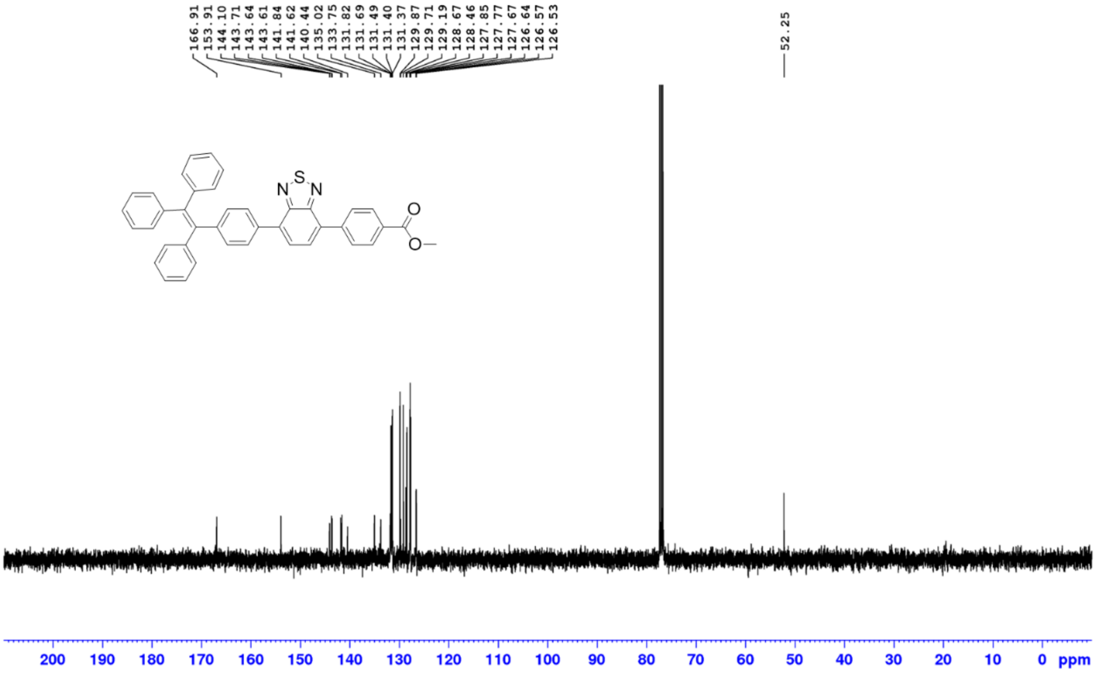

Figure S4. ^{13}C NMR spectrum ($CDCl_3$, 100 MHz, 298 K) of compound **2**.

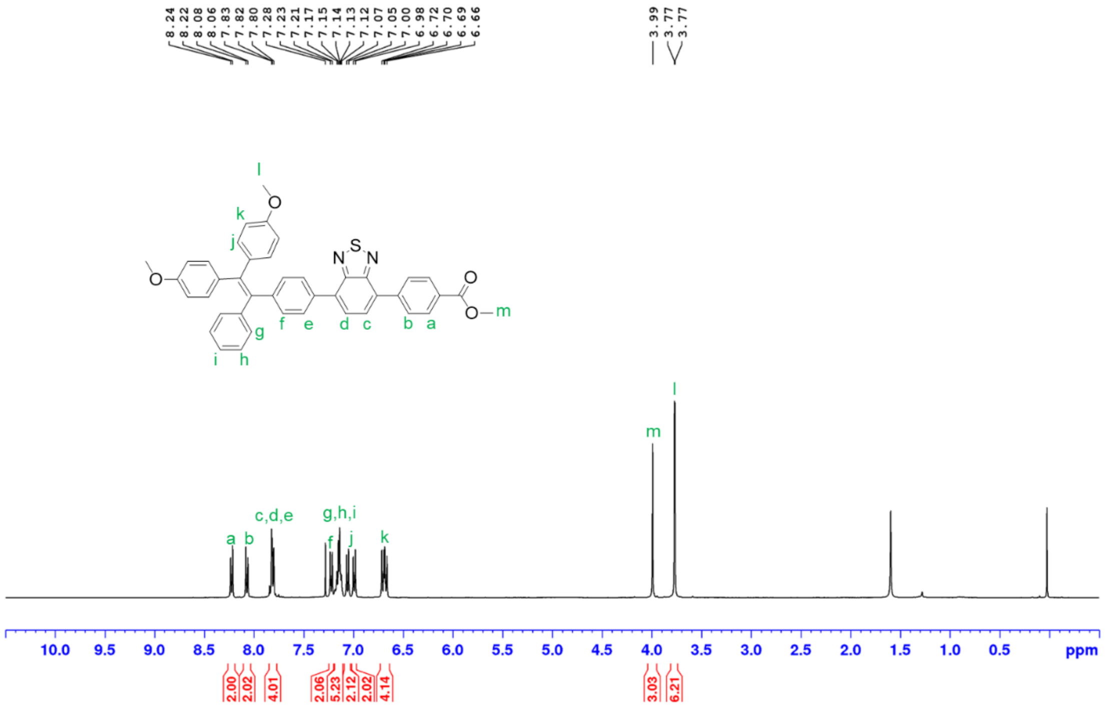

Figure S5. ^{1}H NMR spectrum ($CDCl_3$, 400 MHz, 298 K) of compound **3**.

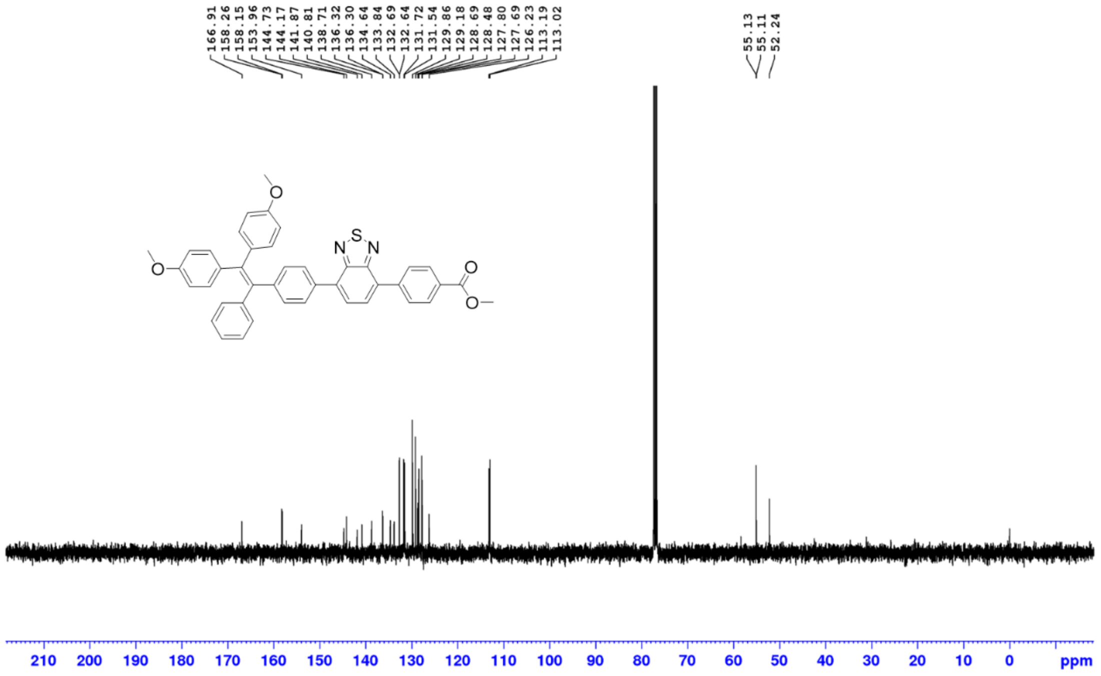

Figure S6. ^{13}C NMR spectrum ($CDCl_3$, 100 MHz, 298 K) of compound **3**.

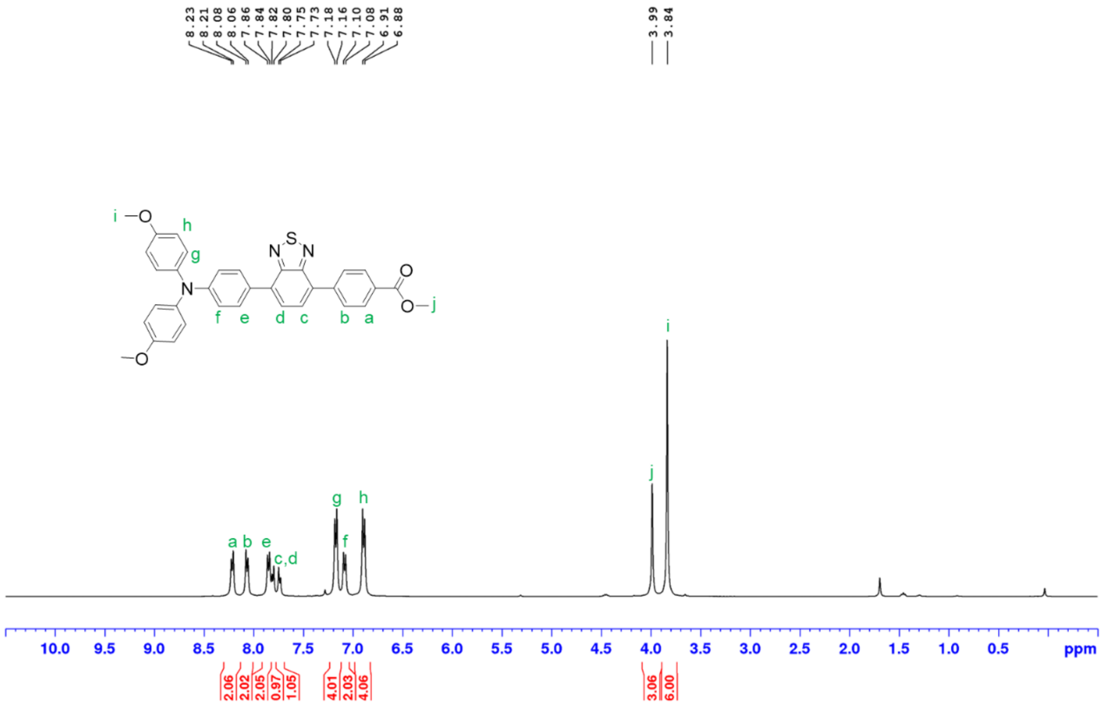

Figure S7. ^{1}H NMR spectrum ($CDCl_3$, 400 MHz, 298 K) of compound **4**.

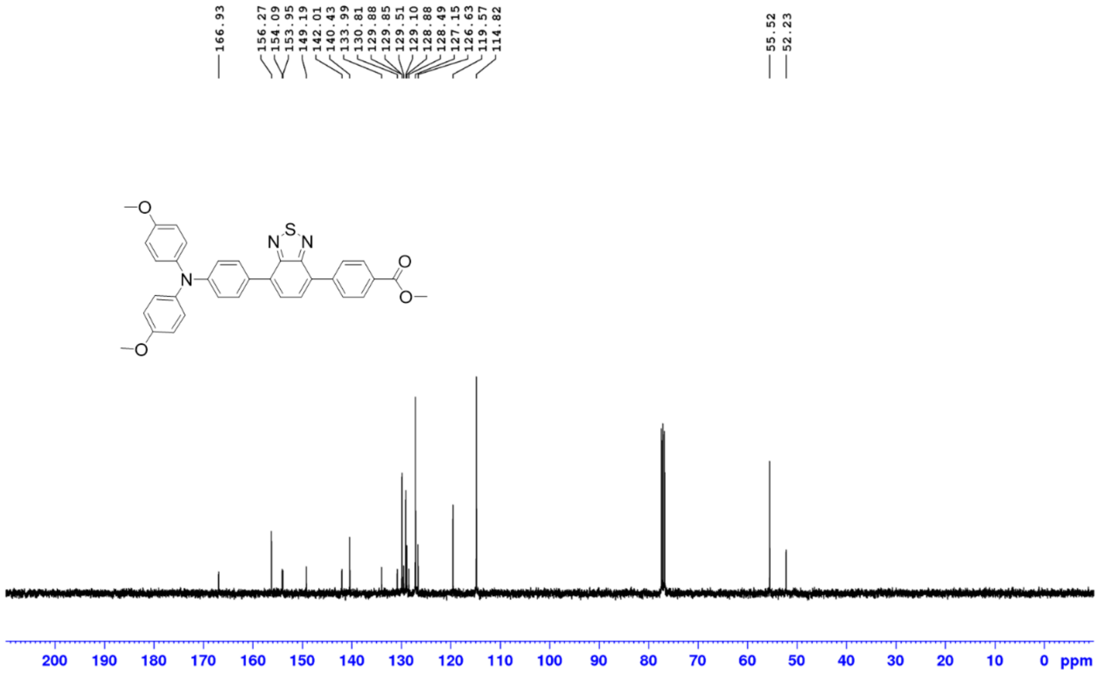

Figure S8. ^{13}C NMR spectrum ($CDCl_3$, 100 MHz, 298 K) of compound **4**.

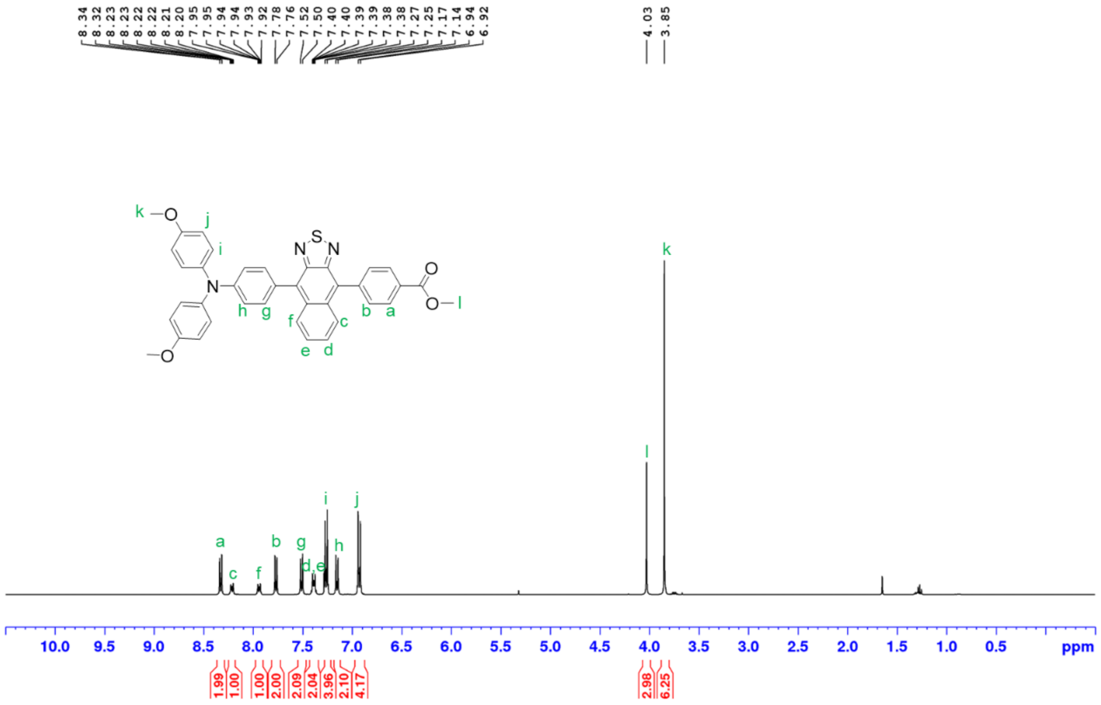

Figure S9. ^{1}H NMR spectrum (CDCl$_3$, 400 MHz, 298 K) of compound **5**.

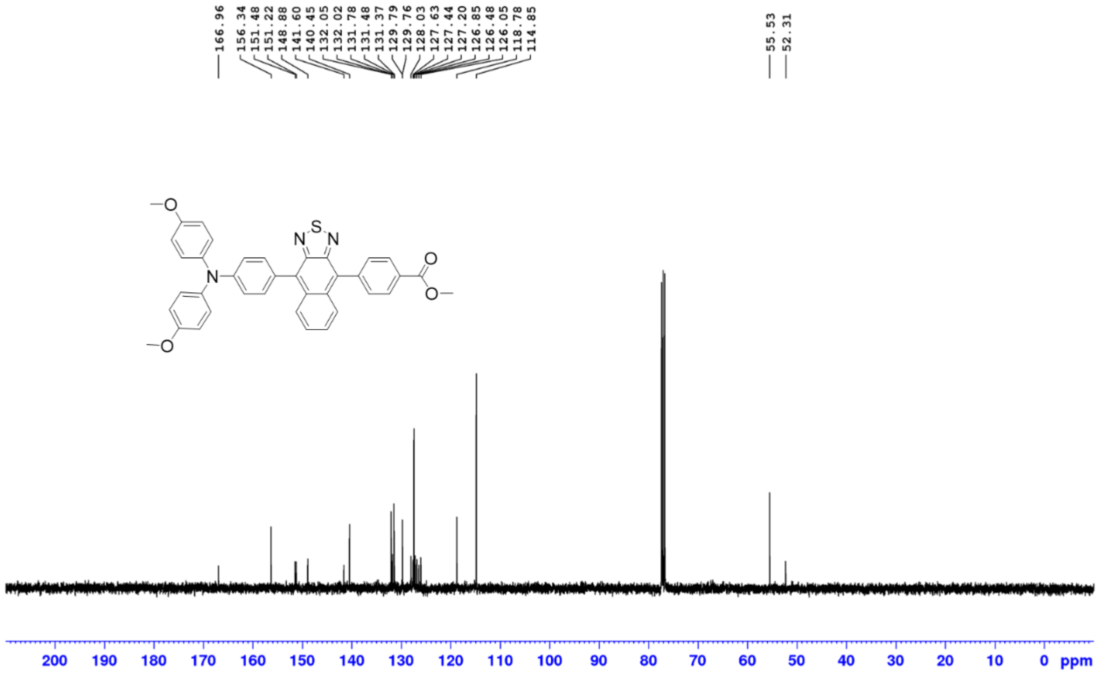

Figure S10. ^{13}C NMR spectrum (CDCl$_3$, 100 MHz, 298 K) of compound **5**.

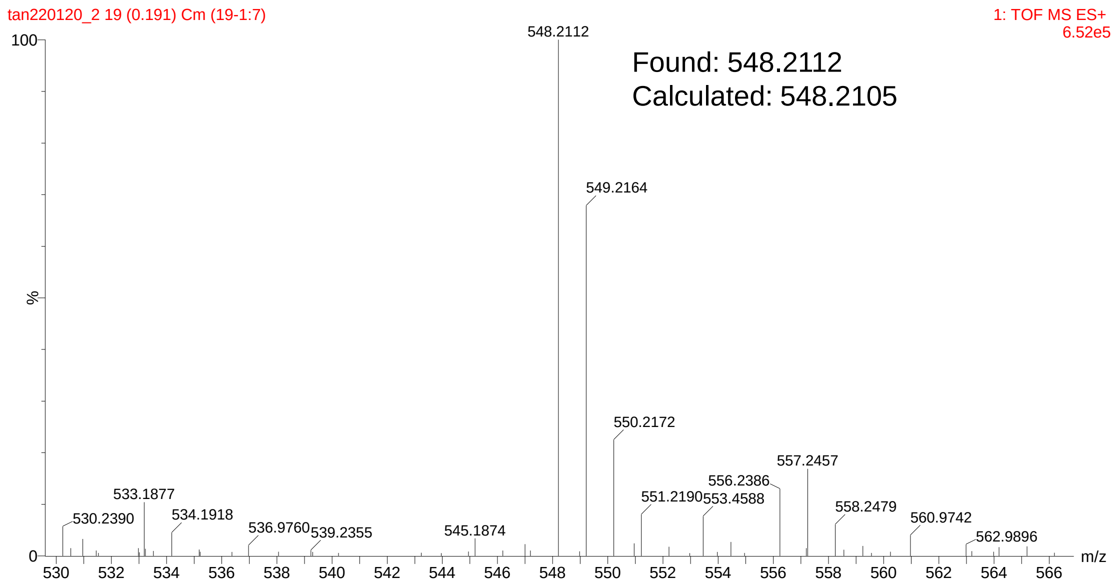

Figure S11. MALDI-TOF mass spectrum of B-tp.

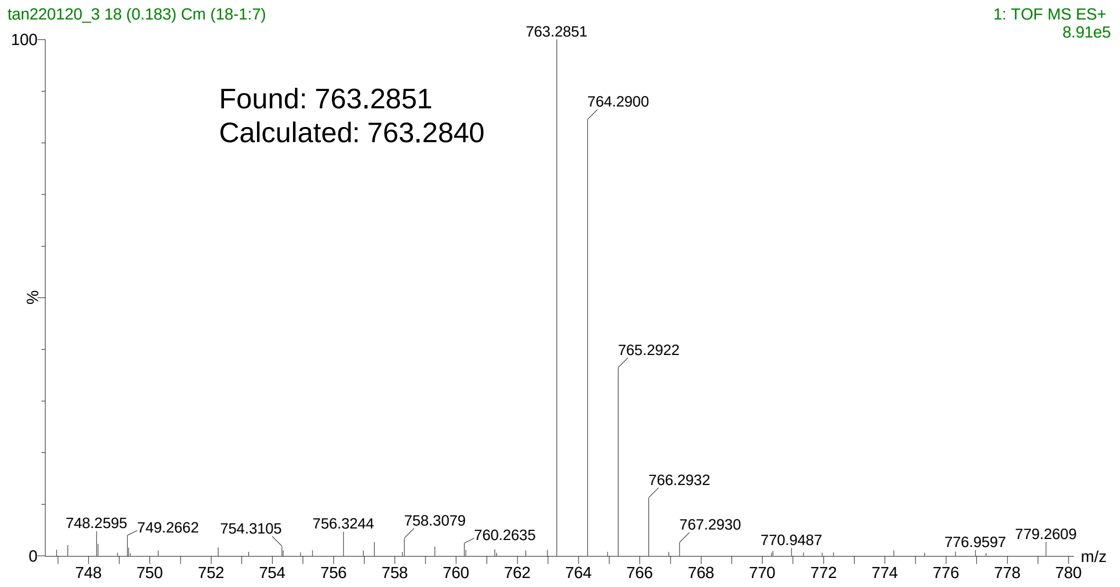

Figure S12. MALDI-TOF mass spectrum of G-tp.

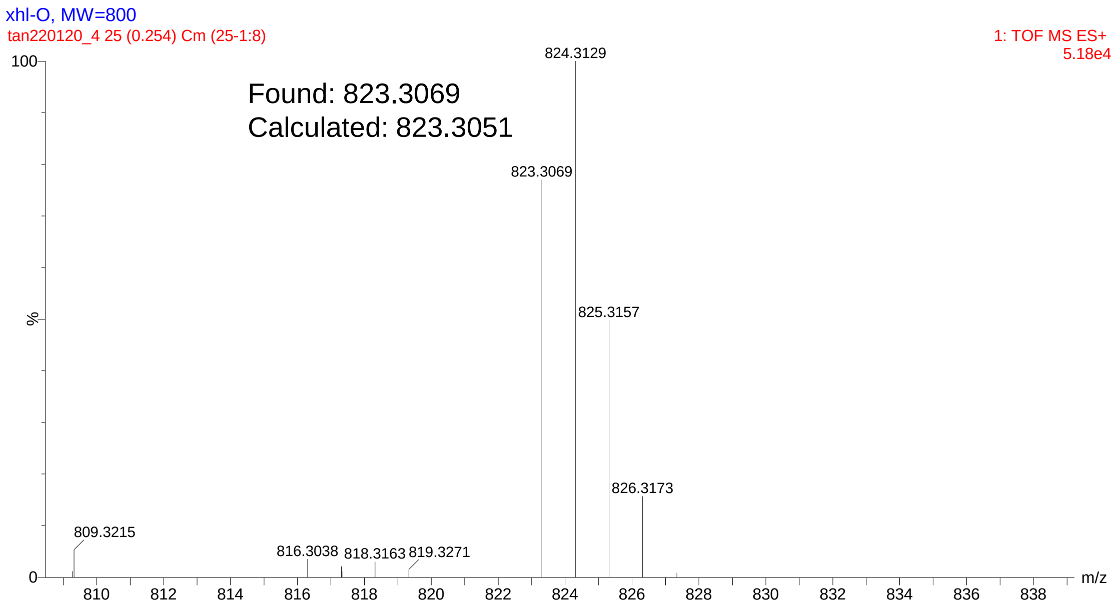

Figure S13. MALDI-TOF mass spectrum of Y-tp.

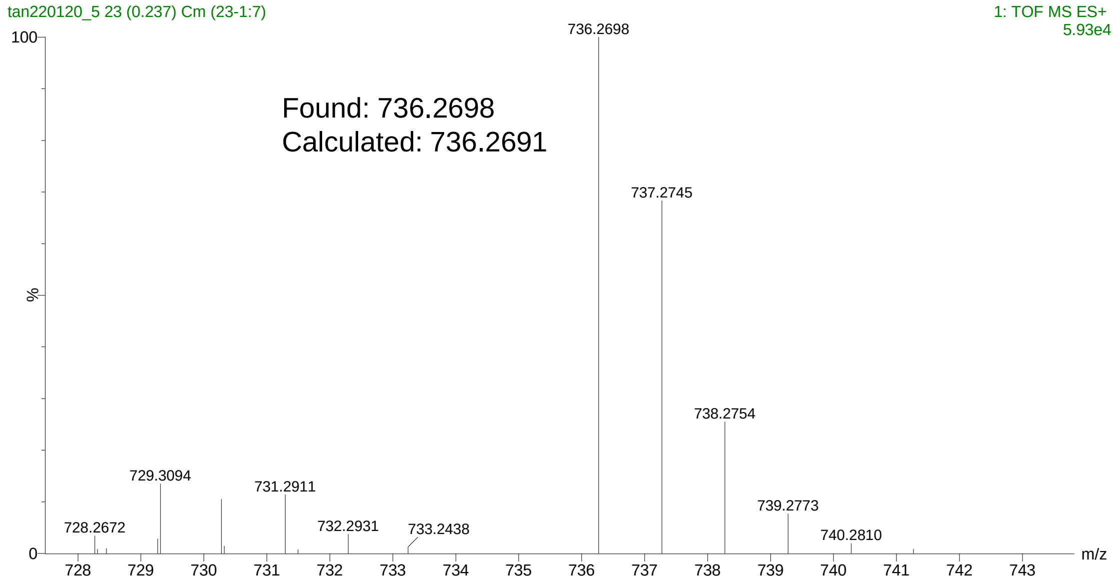

Figure S14. MALDI-TOF mass spectrum of O-tp.

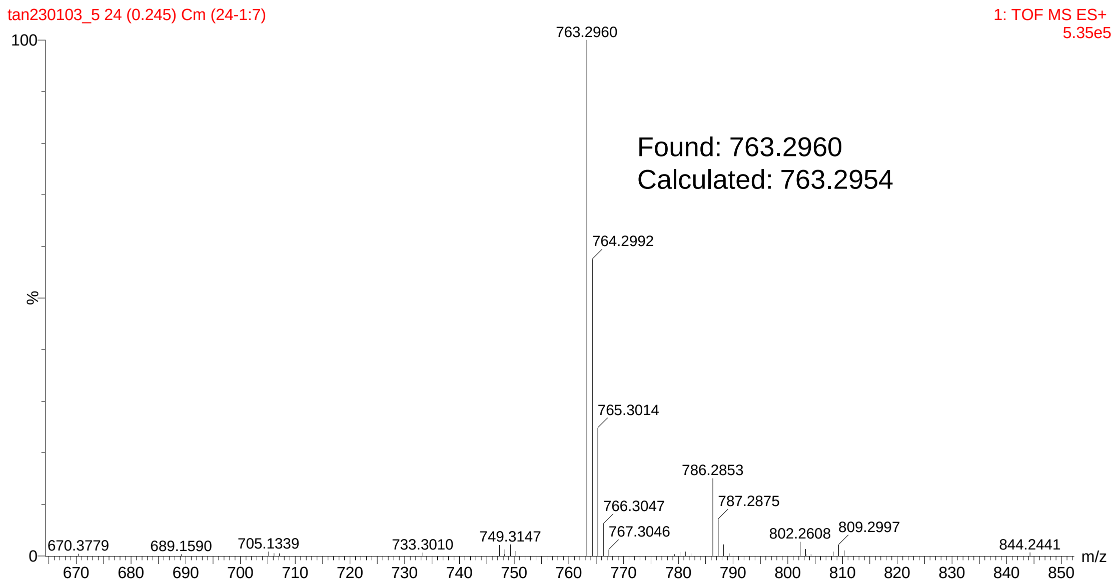

Figure S15. MALDI-TOF mass spectrum of R-tp.

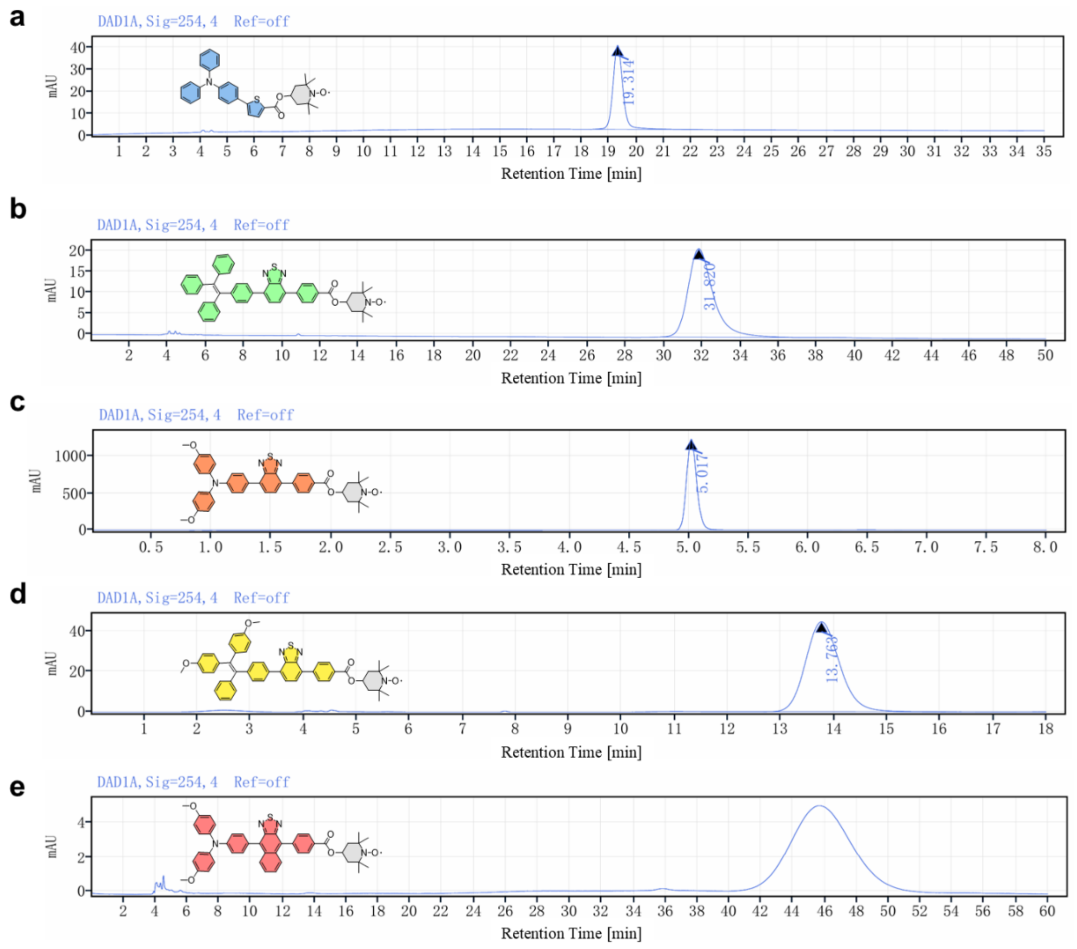

Figure S16. High-performance liquid chromatography (HPLC) results of the TEMPO derivatives: a) B-tp, b) G-tp, c) Y-tp, d) O-tp, and e) R-tp.

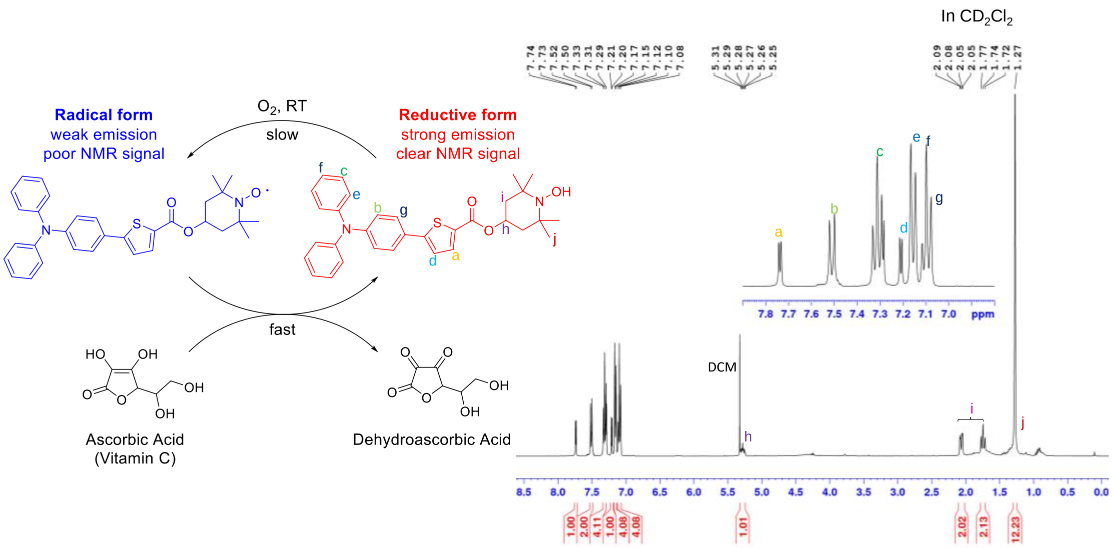

Figure S17. Left: reaction scheme of B-tp with ascorbic acid to generate B-tp-H; right: ^{1}H NMR spectrum (CDCl3, 400 MHz, 298 K) of B-tp-H.

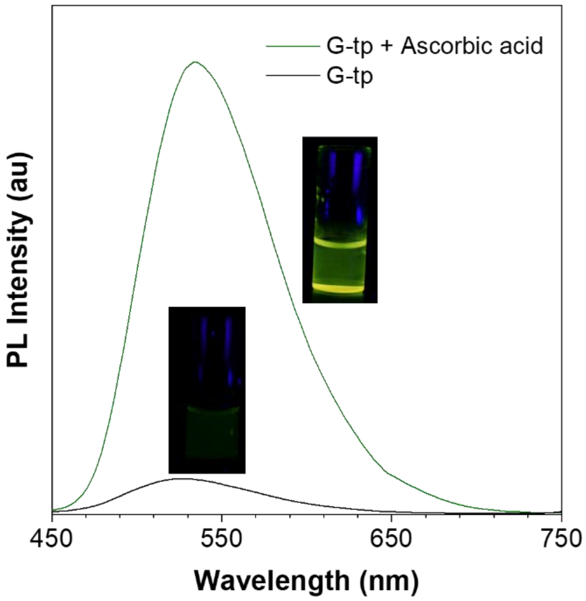

Figure S18. PL spectra of G-tp in THF/water mixture (1: 9 vol %) and the turn-on fluorescence after adding ascorbic acid in the same water fraction. (Insert: photos shot before and after adding ascorbic acid to the G-tp solution)

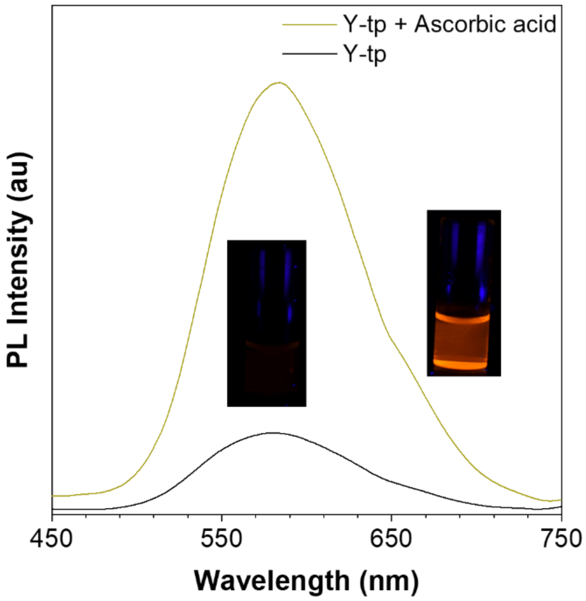

Figure S19. PL spectra of Y-tp in THF/water mixture (1: 9 vol %) and the turn-on fluorescence after adding ascorbic acid in the same water fraction. (Insert: photos shot before and after adding ascorbic acid to the Y-tp solution)

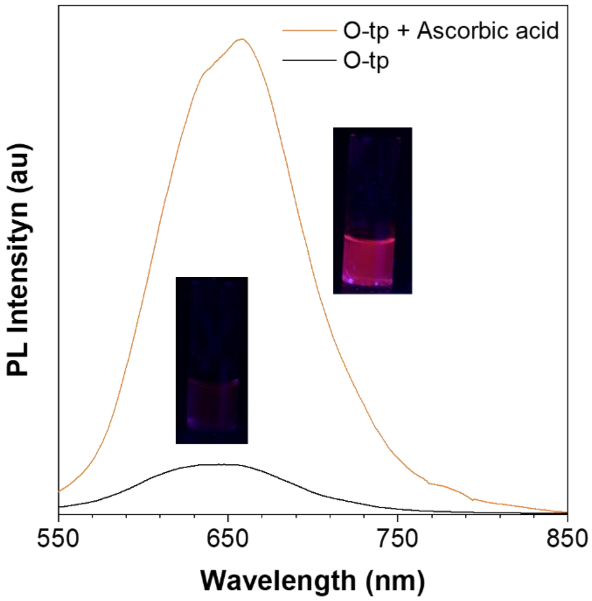

Figure S20. PL spectra of O-tp in THF/water mixture (1: 9 vol %) and the turn-on fluorescence after adding ascorbic acid in the same water fraction. (Insert: photos shot before and after adding ascorbic acid to the O-tp solution)

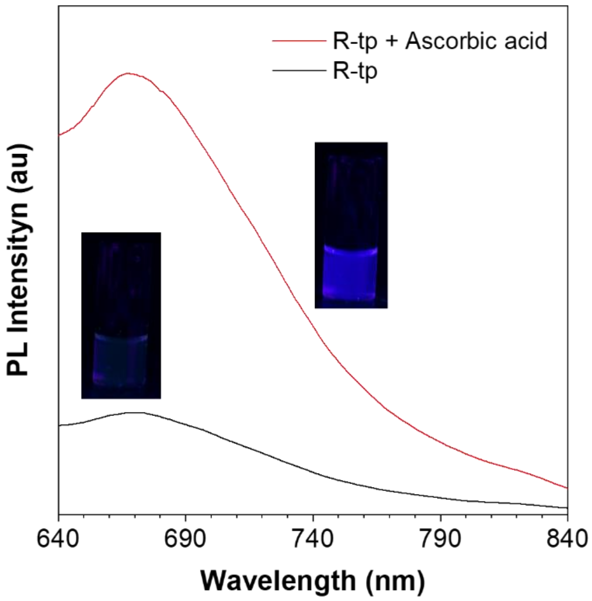

Figure S21. PL spectra of R-tp in THF/water mixture (1: 9 vol %) and the turn-on fluorescence after adding ascorbic acid in the same water fraction. (Insert: photos shot before and after adding ascorbic acid to the R-tp solution)

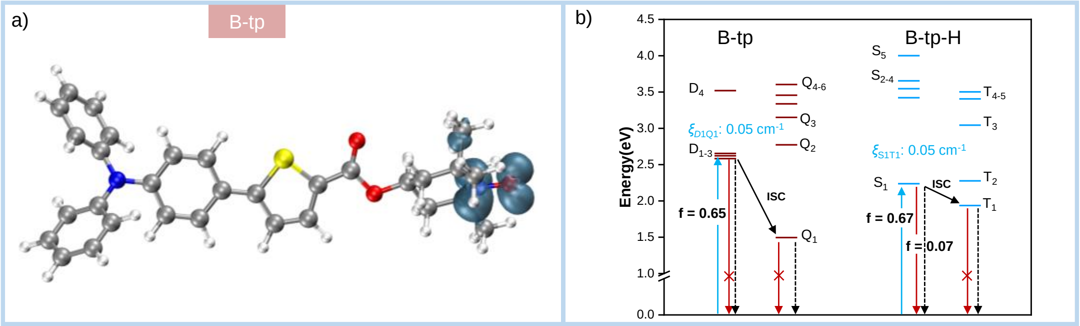

Figure 22. a) Spin density plots for B-tp molecule. Blue/red regions indicate the areas of small spin polarization. b) Calculated energy levels with quartet and triplet states for distinct luminescence behaviors of the B-tp-H and B-tp molecules. Blue arrows correspond to absorption, red arrows correspond to emission and black arrows correspond to the nonradiative (Non-rad.) quenching processes.

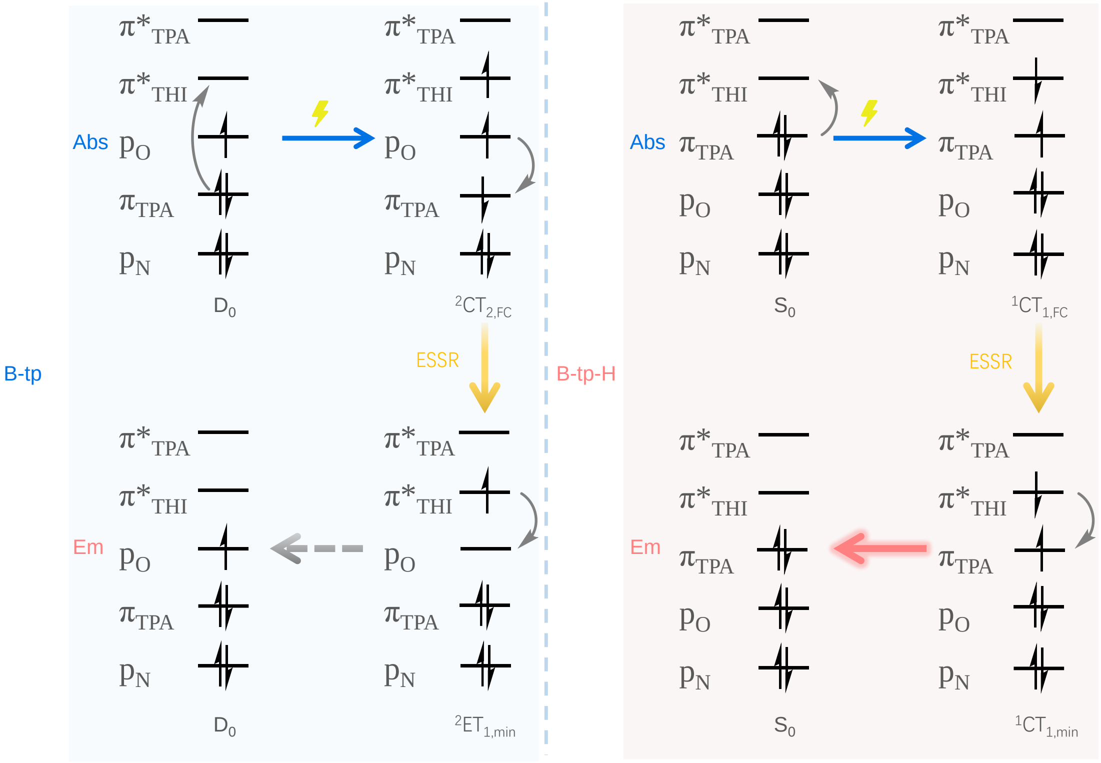

Figure 23. FMO energy diagrams of deactivation mechanism. The ESSR denotes excites state structure relaxation.

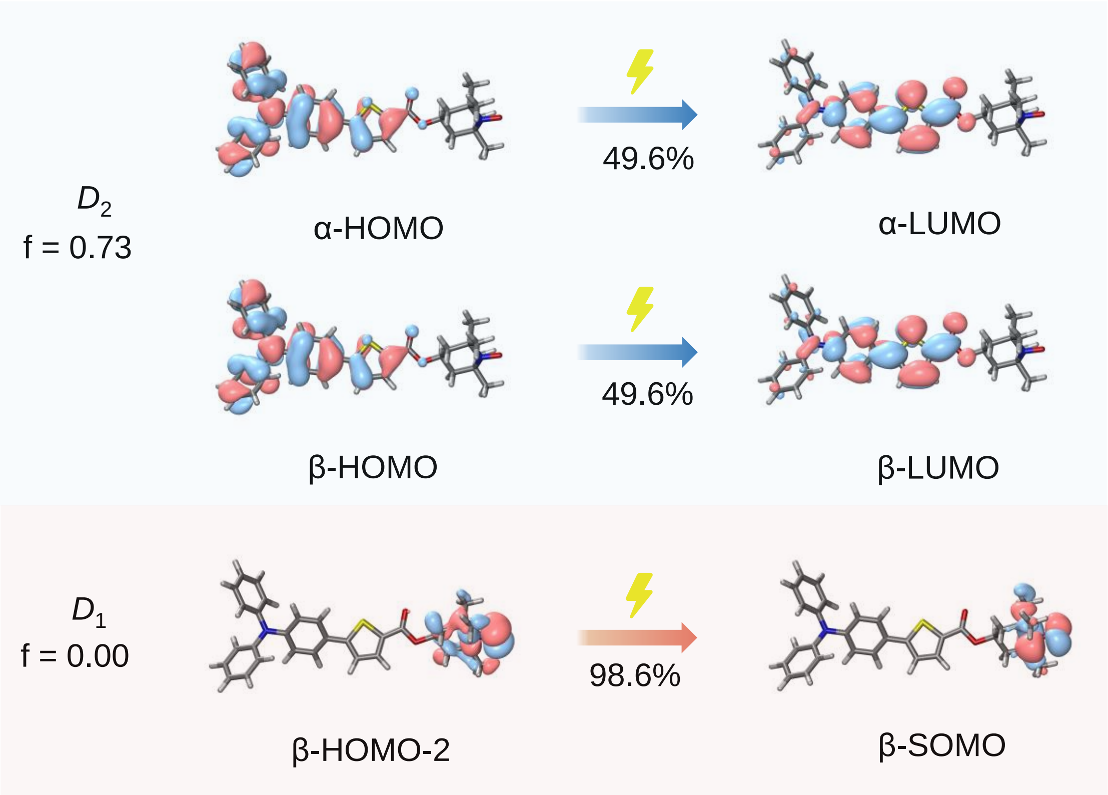

Figure 24. Molecular orbitals for the corresponding electronic transitions of B-tp at the optimized ground D_0 state conformation. of the B-tp at ground state.

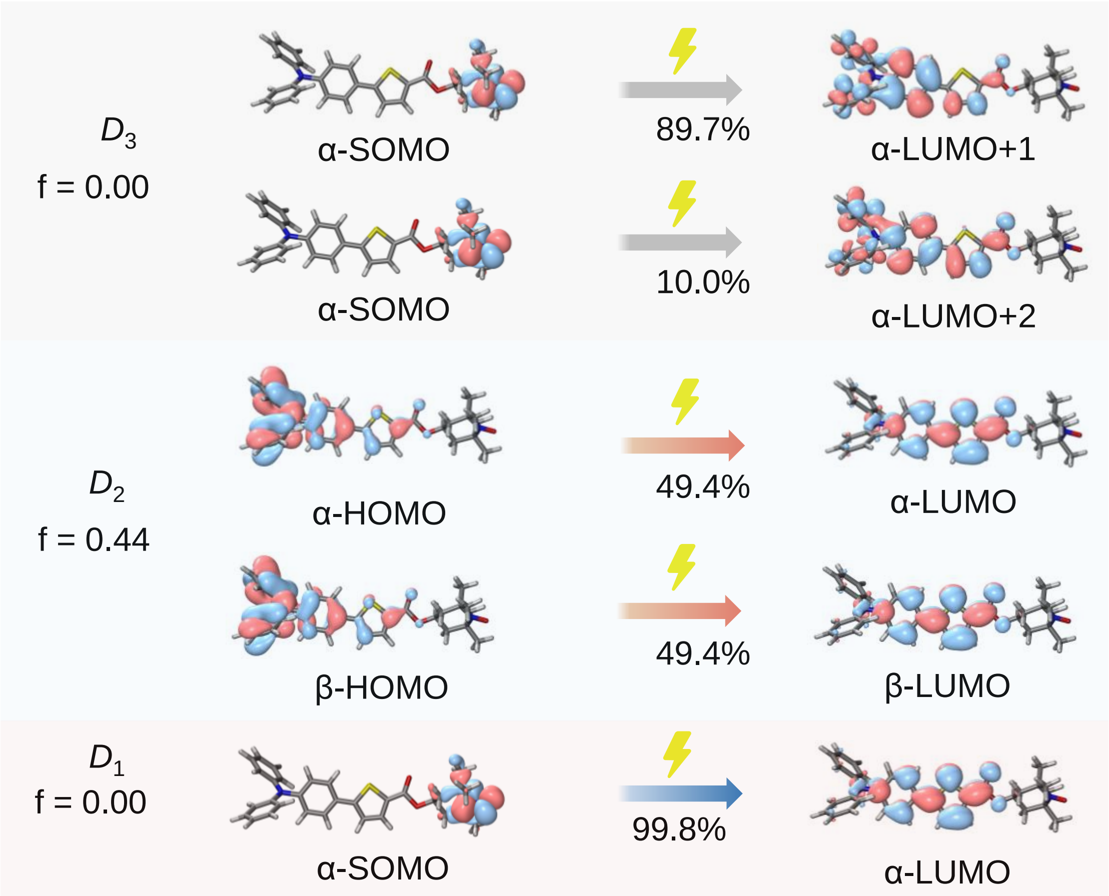

Figure 25. Molecular orbitals for the corresponding electronic transitions of B-tp at the optimized excited D_1 state conformation.

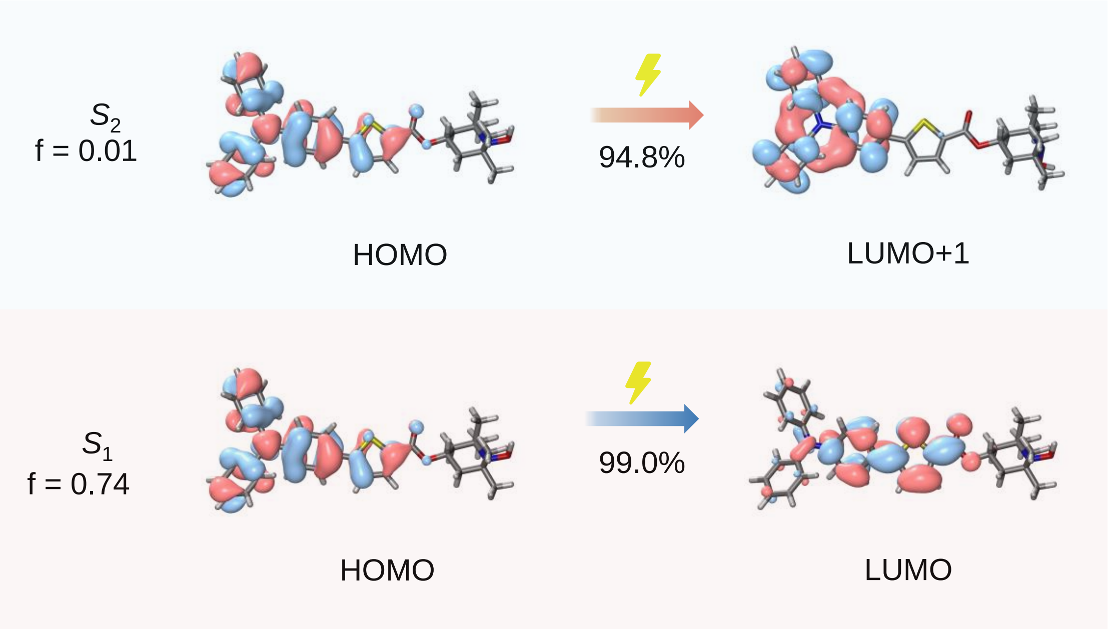

Figure 26. Molecular orbitals for the corresponding electronic transitions of B-tp-H at the ground S_0 state conformation.

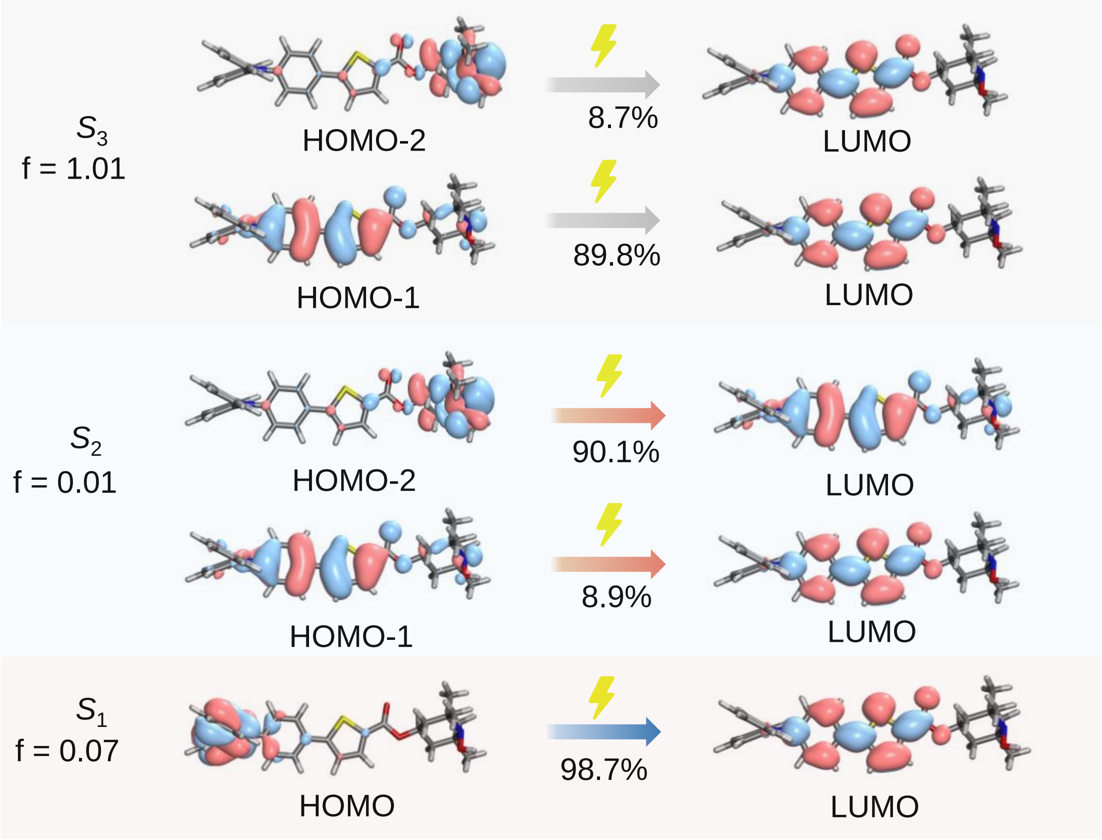

Figure 27. Molecular orbitals for the corresponding electronic transitions of B-tp-H at the optimized excited S_1 state conformation.

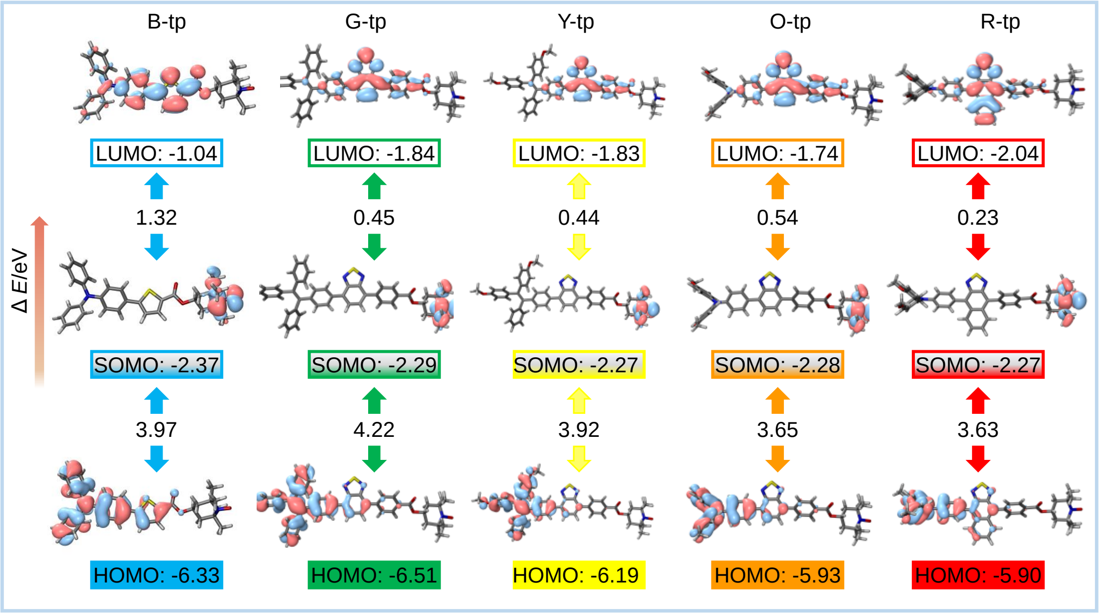

Figure S28. Optimized S_0 geometries and illustration of the frontier molecular orbitals (LUMOs and HOMOs) determined by the B3LYP/6-31G* level of theory.

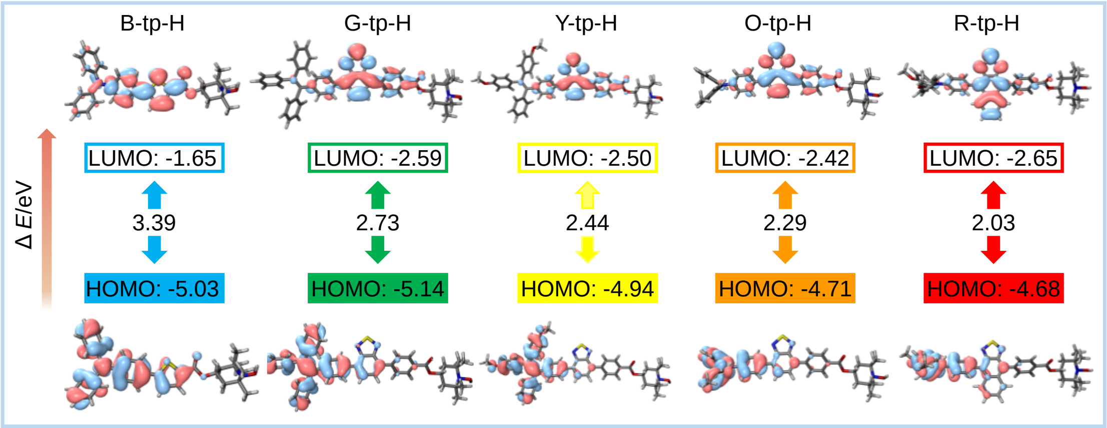

Figure S29. Optimized S_0 geometries and illustration of the frontier molecular orbitals (LUMOs and HOMOs) determined by the B3LYP/6-31G* level of theory.

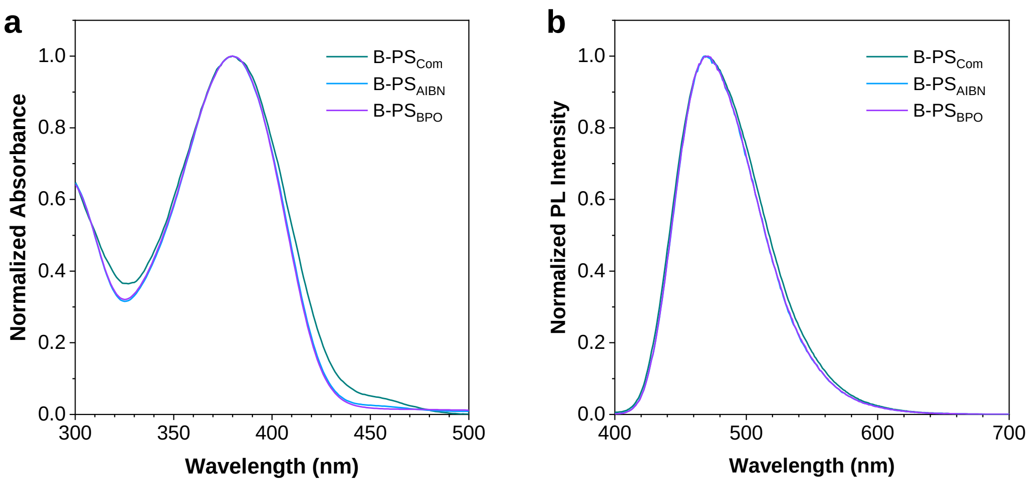

Figure S30. a) Absorption and b) emission spectra of fluorescent B-PSs obtained by PS samples from different synthetic methods (AIBN and BPO as initiators for free-radical polymerization)

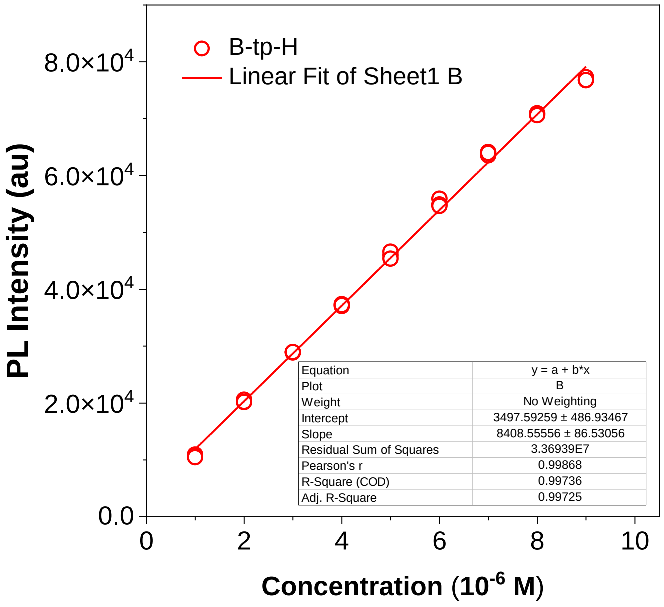

Figure S31. Calibration curve for the fluorescence intensity of B-tp-H as a function of its concentration in dilute toluene.

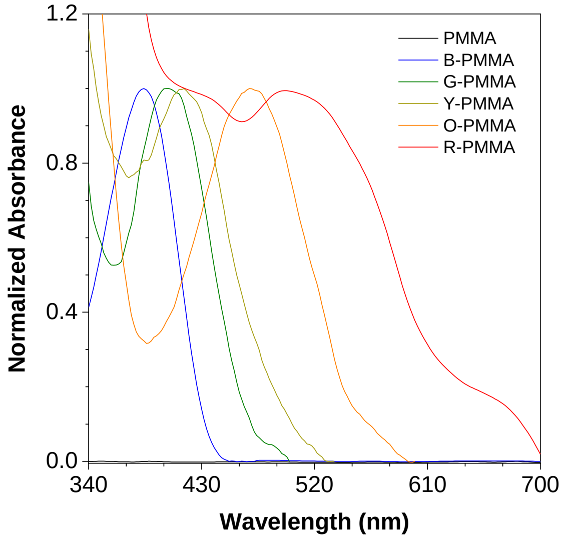

Figure S32. Normalized absorbance spectra of pure PMMA, and the various fluorescent PMMA (PMMA-B, PMMA-G, PMMA-Y, PMMA-O, and PMMA-R) in the solid state.

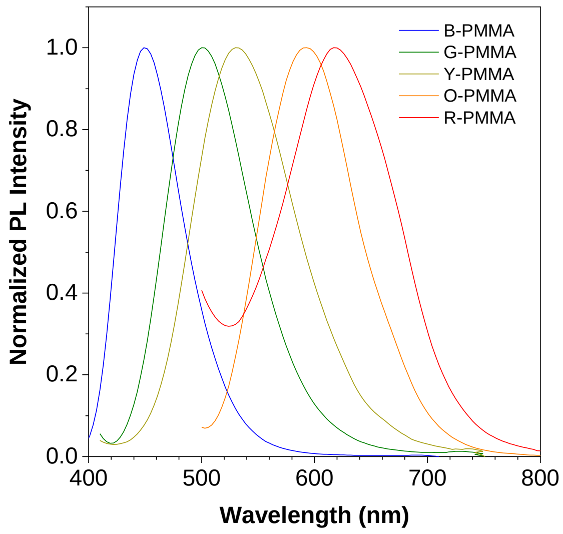

Figure S33. Normalized PL spectra of the obtained fluorescent PMMA.

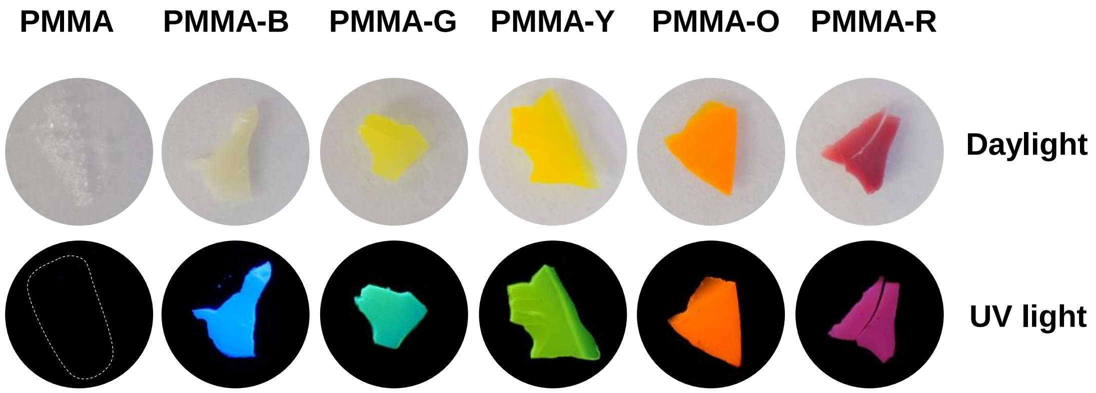

Figure S34. Photographs of PMMA and AIE pre-fluorophores-attached PMMA under daylight and UV light.

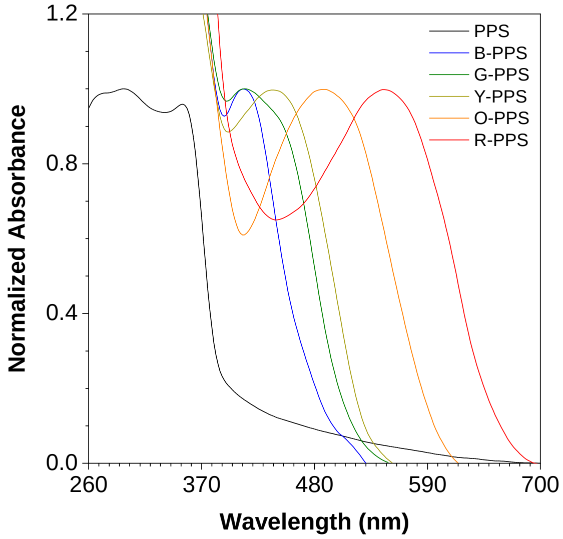

Figure S35. Normalized absorbance spectra of pure PPS, and the various fluorescent PPS (PPS-B, PPS-G, PPS-Y, PPS-O, and PPS-R) in the solid state.

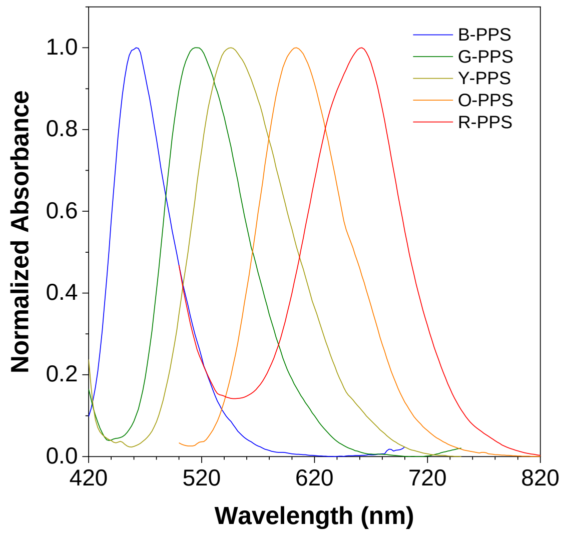

Figure S36. Normalized PL spectra of the obtained fluorescent PPS.

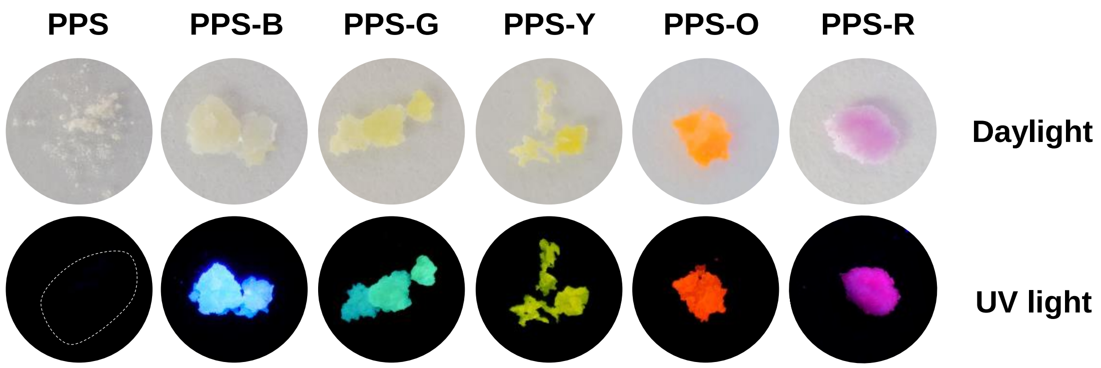

Figure S37. Photographs of PPS and AIE pre-fluorophores-attached PPS under daylight and UV light.

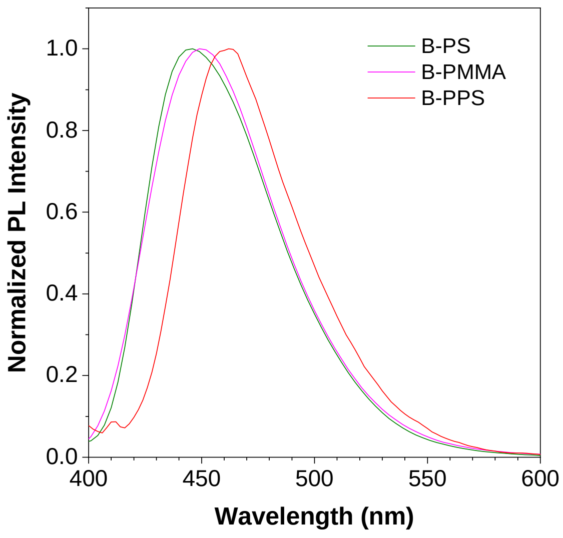

Figure S38. Normalized PL spectra of the obtained B-tp modified polymers.

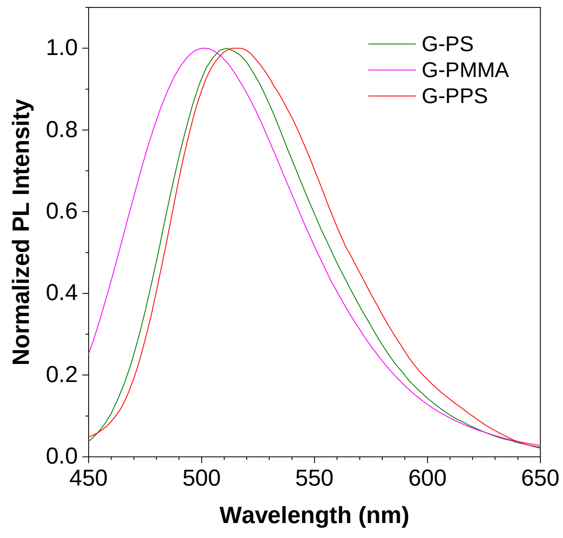

Figure S39. Normalized PL spectra of the obtained G-tp modified polymers.

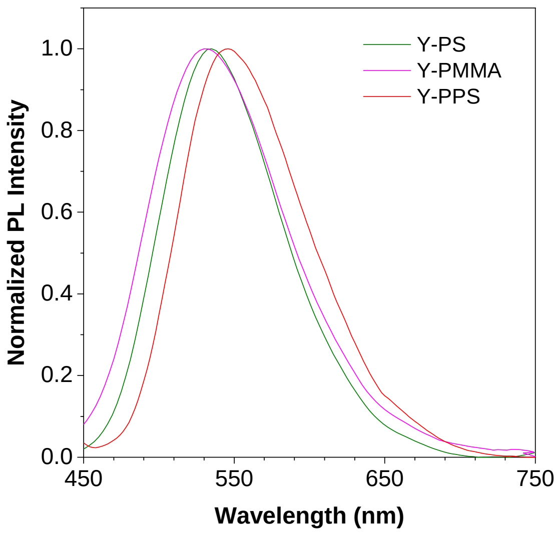

Figure S40. Normalized PL spectra of the obtained Y-tp modified polymers.

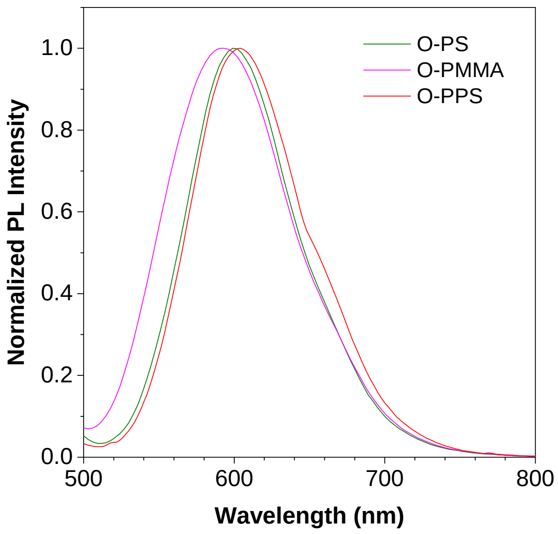

Figure S41. Normalized PL spectra of the obtained O-tp modified polymers.

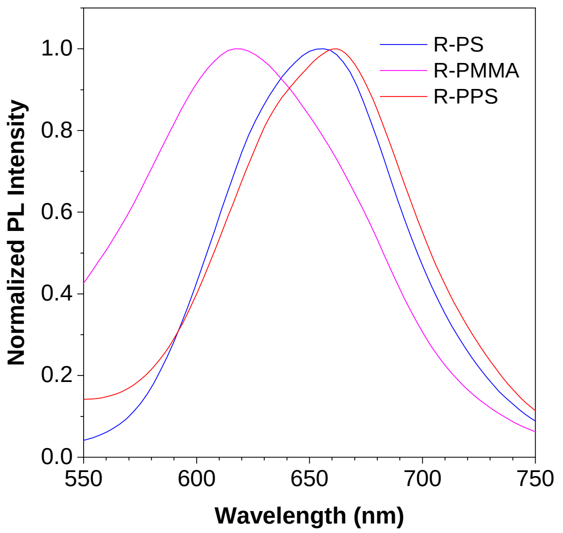

Figure S42. Normalized PL spectra of the obtained R-tp modified polymers.

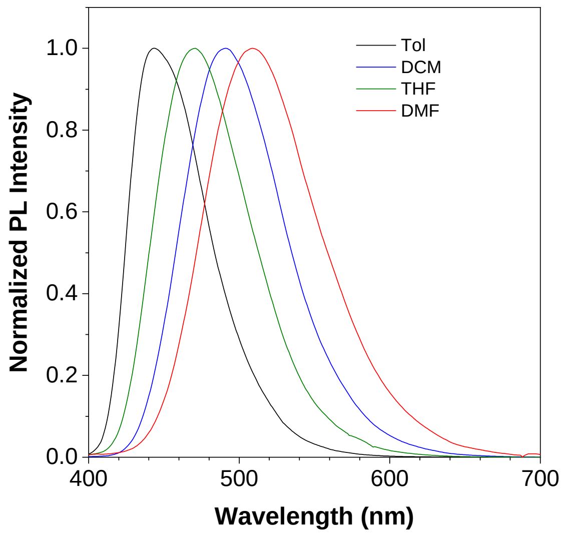

Figure S43. Normalized PL spectra of the obtained fluorescent B-PS in different solvents (Tol: Toluene; DCM: Dichloromethane; THF: Tetrahydrofuran; DMF: Dimethylformamide)

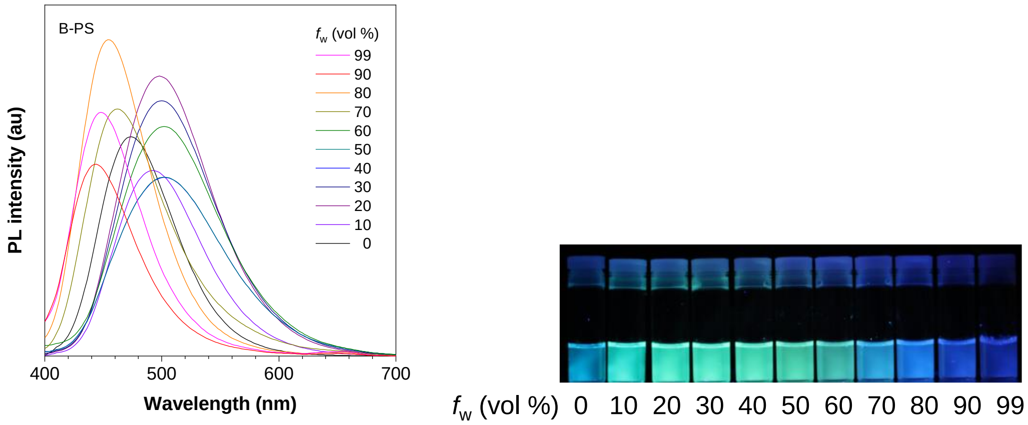

Figure S44. Left: PL spectra of B-PS in THF/H_2O mixture with different water fractions (f_w). Right: photos shot under different water fractions.

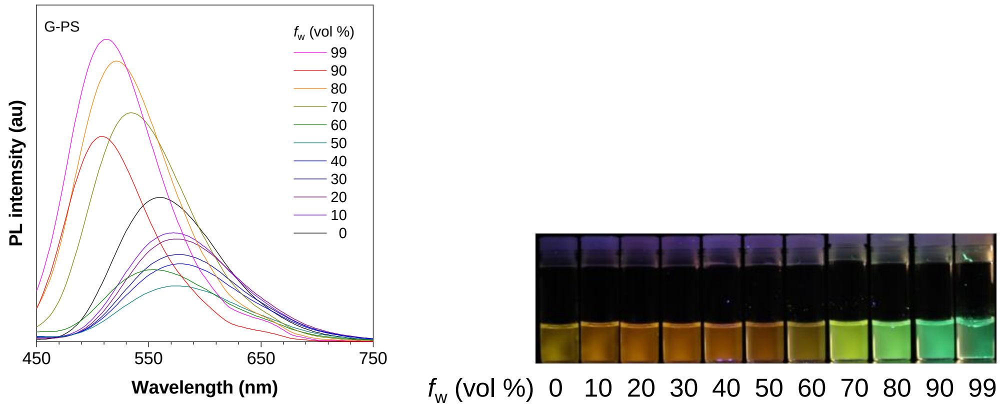

Figure S45. Left: PL spectra of G-PS in THF/H_2O mixture with different water fractions (f_w). Right: photos shot under different water fractions.

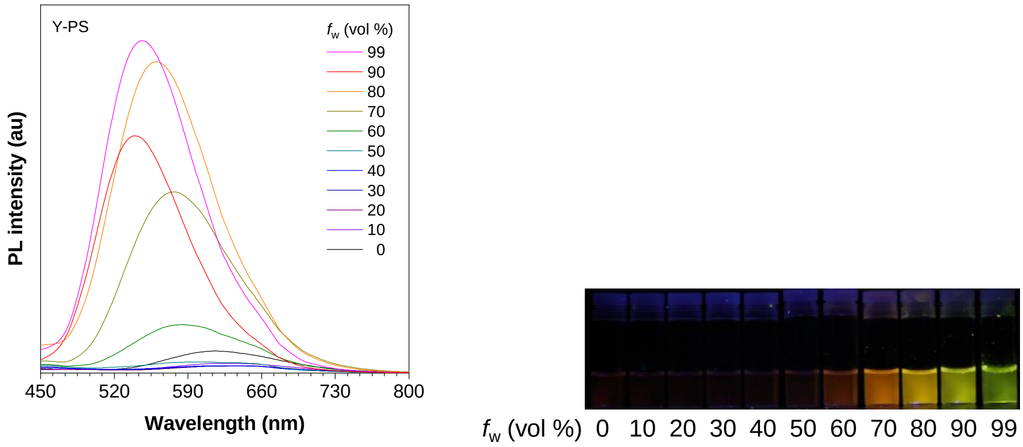

Figure S46. Left: PL spectra of Y-PS in THF/H_2O mixture with different water fractions (f_w). Right: photos shot under different water fractions.

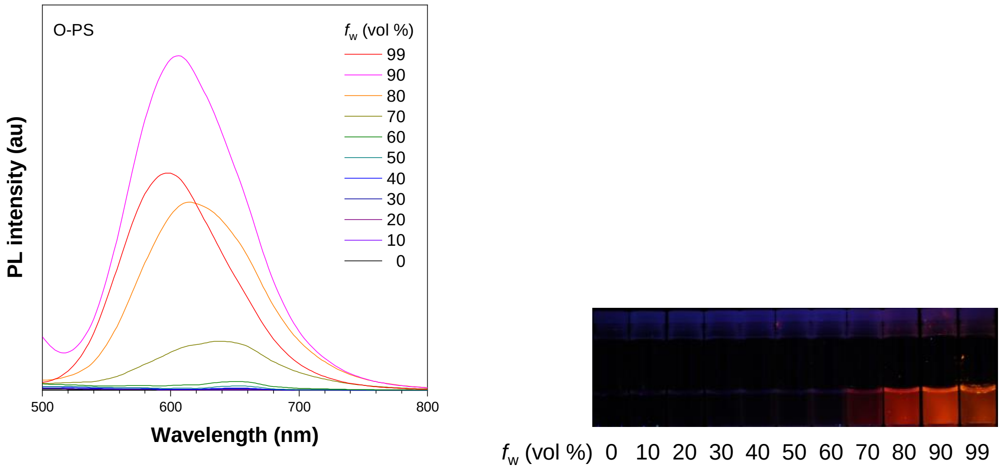

Figure S47. Left: PL spectra of O-PS in THF/H_2O mixture with different water fractions (f_w). Right: photos shot under different water fractions.

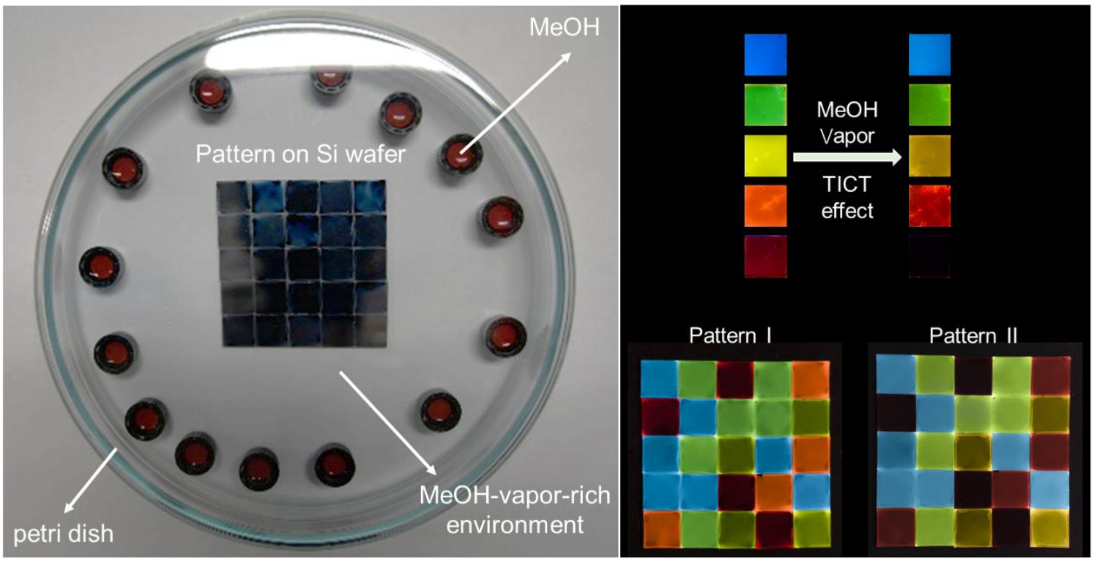

Figure S48. Photographs of the specific experimental conditions when placing the array with methanol fumigation under daylight.

Table S1. M_n and PDI of polymer samples before and after ball milling in the presence of AIE pre-fluorophores

Compound	M_n [a]	M_w/M_n [a]
PS	79,200	1.02
B-PS	16,700	1.24
G-PS	17,600	1.24
Y-PS	17,500	1.24
O-PS	17,300	1.25
R-PS	10,000	1.25

[a] Determined by GPC in THF, based on linear PS as a calibration standard.

Table S2. The amount of dye incorporated within the polymer after ball milling.

	Dye Amount (µmol/mg)	Incorporation Rate (%wt)
B-PS	0.0285	1.5
G-PS	0.0321	1.6
Y-PS	0.0229	1.9
O-PS	0.0256	1.9
R-PS	0.0202	1.5

Table S3. Photoluminescence quantum yield (PLQY) of the AIE pre-fluorophores and the AIE pre-fluorophores-attached polymers

Compound	B-tp	G-tp	Y-tp	O-tp	R-tp
Tp	~ 0.0	~ 0.5	~ 0.7	~ 0.0	~ 0.2
PS	26.3	40.8	53.0	40.6	47.9
PMMA	47.8	54.0	54.1	50.5	9.2
PPS	12.1	4.7	6.0	20.5	7.5

Table S4 Photophysical parameters of AIE pre-fluorophores and the AIE pre-fluorophores-attached polymers

Compound	B-tp	G-tp	Y-tp	O-tp	R-tp	PS	B-PS	G-PS	Y-PS	O-PS	R-PS
λ_{Abs}(nm)[a]	386	413	427	484	546	260	386	412	426	478	542
λ_{Em}(nm)[b]	/	/	/	/	/	/	446	511	535	599	656
τ_F(ns)[c]	/	/	/	/	/	/	0.72	1.66	2.60	6.84	12.98

[a] The wavelength of absorption maximum in the solid state

[b] The wavelength of emission maximum in the solid state

[c] Lifetime determined the solid state at room temperature
